# High Oxygen Barrier Polyester from 3,3′-Bifuran-5,5′-dicarboxylic Acid

**DOI:** 10.1021/acsmacrolett.2c00743

**Published:** 2023-01-13

**Authors:** Tuomo P. Kainulainen, Tomi A. O. Parviainen, Juho Antti Sirviö, Liam J. R. McGeachie, Juha P. Heiskanen

**Affiliations:** † Research Unit of Sustainable Chemistry, University of Oulu, P.O. Box 4300, FI-90014 Oulu, Finland; ‡ Fibre and Particle Engineering Research Unit, University of Oulu, P.O. Box 4300, FI-90014 Oulu, Finland; § Laboratory of Inorganic Chemistry, Environmental and Chemical Engineering, University of Oulu, P.O. Box 4300, FI-90014 Oulu, Finland

## Abstract

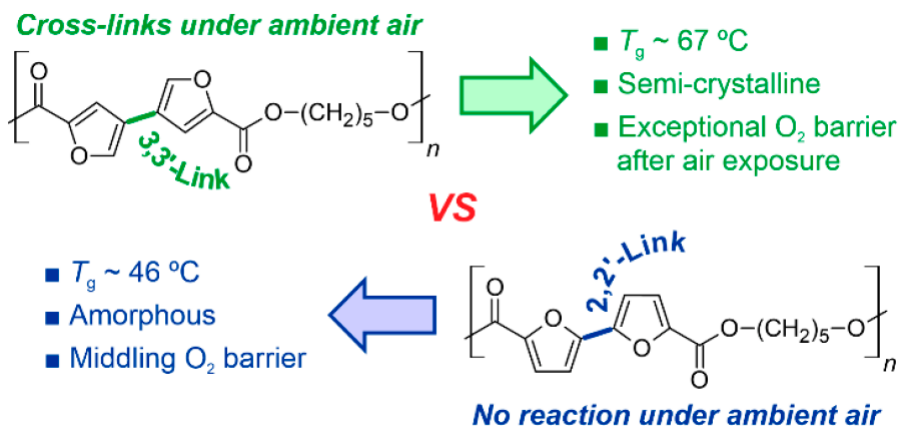

An exceptional oxygen barrier polyester prepared from a new biomass-derived monomer, 3,3′-bifuran-5,5′-dicarboxylic acid, is reported. When exposed to air, the furan-based polyester cross-links and gains O_2_ permeability 2 orders of magnitude lower than initially, resulting in performance comparable to the best polymers in this class, such as ethylene-vinyl alcohol copolymers. The cross-links hinder the crystallization of amorphous samples, also rendering them insoluble. The process was observable via UV–vis measurements, which showed a gradual increase of absorbance between wavelengths of 320 and 520 nm in free-standing films. The structural trigger bringing about these changes appears subtle: the polyester containing 5,5′-disubstituted 3,3′-bifuran moieties cross-linked, whereas the polyester with 5,5′-disubstituted 2,2′-bifuran moieties was inert. The 3,3′-bifuran-based polyester is effectively a semicrystalline thermoplastic, which is slowly converted into a cross-linked material with intriguing material properties once sufficiently exposed to ambient air.

Furan rings as structural units have proven promising in novel and sustainable polymeric materials.^1−3^ Furan precursors used for these purposes are derived from abundant biomasses: these include carbohydrates, which can be converted into 2-furancarboxaldehyde (furfural) and 5-hydroxymethyl-2-furancarboxaldehyde (HMF) via dehydration. Furfural is produced at an industrial scale, and there is considerable interest nowadays toward larger-scale HMF production as well.^4^ HMF serves as a stepping stone toward 2,5-furan dicarboxylic acid (2,5-FDCA), which is applicable as a monomer in polyesters,^5−7^ polyamides,^8−10^ and others,^11,12^ especially as a replacement for traditional monomers such as terephthalic acid. Furfural is largely converted into furfuryl alcohol, a precursor for resins, although it also represents a platform with potential for a wide variety of products.^13,14^ To achieve 2,5-FDCA-like uses in polymers, monofunctional furfural must be modified. For instance, a dicarboxylic acid may be obtained by coupling furfural or its derivatives into difunctional bifurans.^15,16^ Other strategies have included photochemical reactions of derivatives and reactions with bridge-forming electrophiles (formaldehyde, acetone) to obtain dicarboxylic acids or esters.^17−19^ We also recently described the application of sulfur atoms in this bridging role.^20^

The variation of the furan ring substitution pattern appears to be a useful tool for modifying the properties of subsequent polymers.^21^ For example, poly(butylene-2,4-furanoate) (2,4-PBF) reportedly has a far lower O_2_ transmission rate than poly(butylene-2,5-furanoate) (2,5-PBF).^22^ However, the less symmetric 2,4-FDCA moiety of 2,4-PBF also decreased the glass transition temperature (*T*_g_) and crystallinity compared to 2,5-PBF. For bifuran-type monomers, several isomers are possible as well, and they may be accessed via heteroaromatic coupling reactions of furfural derivatives. So far, the only compound in this family that has been studied as a monomer in polyester syntheses is 2,2′-bifuran-5,5′-dicarboxylic acid (2,2′-BFDCA).^15,16^ Therefore, the impact of bifuran isomerism on properties of, e.g., polyesters, has remained uncharted. In this study, a new isomer, 3,3′-bifuran-5,5′-dicarboxylic acid (3,3′-BFDCA), is used as a precursor to synthesize a novel polyester, poly(pentamethylene-3,3′-bifuranoate) (3,3′-PPeBf). It is then compared to its analogue, poly(pentamethylene-2,2′-bifuranoate) (2,2′-PPeBf), in turn prepared from 2,2′-BFDCA. Surprisingly, the results show 3,3′-PPeBf to be an unusual polyester that undergoes slow reaction under air at ambient conditions. The reaction with air results in cross-linking, giving rise to multiple changes in properties. Most notably, the O_2_ gas barrier of film specimens enhanced by 2 orders of magnitude upon air exposure, giving 3,3′-PPeBF properties that compare favorably with known, best-performing O_2_ barrier polymers.

Details related to the experimental work are presented in the Supporting Information. The synthesis route, starting from the commercially available methyl 4-bromo-2-furoate (**1**), is illustrated in Scheme 1a. The homocoupling of bromide **2** into 3,3′-BFDCA (**3**) was accomplished using a modified version of the protocol previously used by Lei et al.^23^ to prepare 2,2′-BFDCA. For the purposes of polyester synthesis, 3,3′-BFDCA was esterified in refluxing methanol using sulfuric acid as the catalyst, yielding the dimethyl ester (**4**). The diester has a high melting point (*T*_m_ = 235 °C) and relatively low solubility in common solvents (e.g., alcohols, DMSO, chloroform). Still, dimethyl ester **4** reacted well with 1,5-pentanediol under the classical transesterification/polycondensation conditions, yielding the desired polyester, 3,3′-PPeBf. Unlike the sparingly soluble monomer, the polyester dissolved easily in typical polyester solvents (e.g., 1,1,1,3,3,3-hexafluoroisopropanol or mixtures of chloroform and trifluoroacetic acid). Intrinsic viscosity [η], measured in phenol/1,1,2,2-tetrachloroethane (60:40 w/w), was 0.97 dL/g. For comparison, 2,2′-PPeBf was prepared using the same four reaction steps starting from methyl 5-bromo-2-furoate (Scheme 1b), yielding a polyester with [η] = 1.03 dL/g. ^1^H NMR measurements confirmed the expected chemical structures of 3,3′-PPeBf and 2,2′-PPeBf (Figures S7 and S8).

**Scheme 1:**
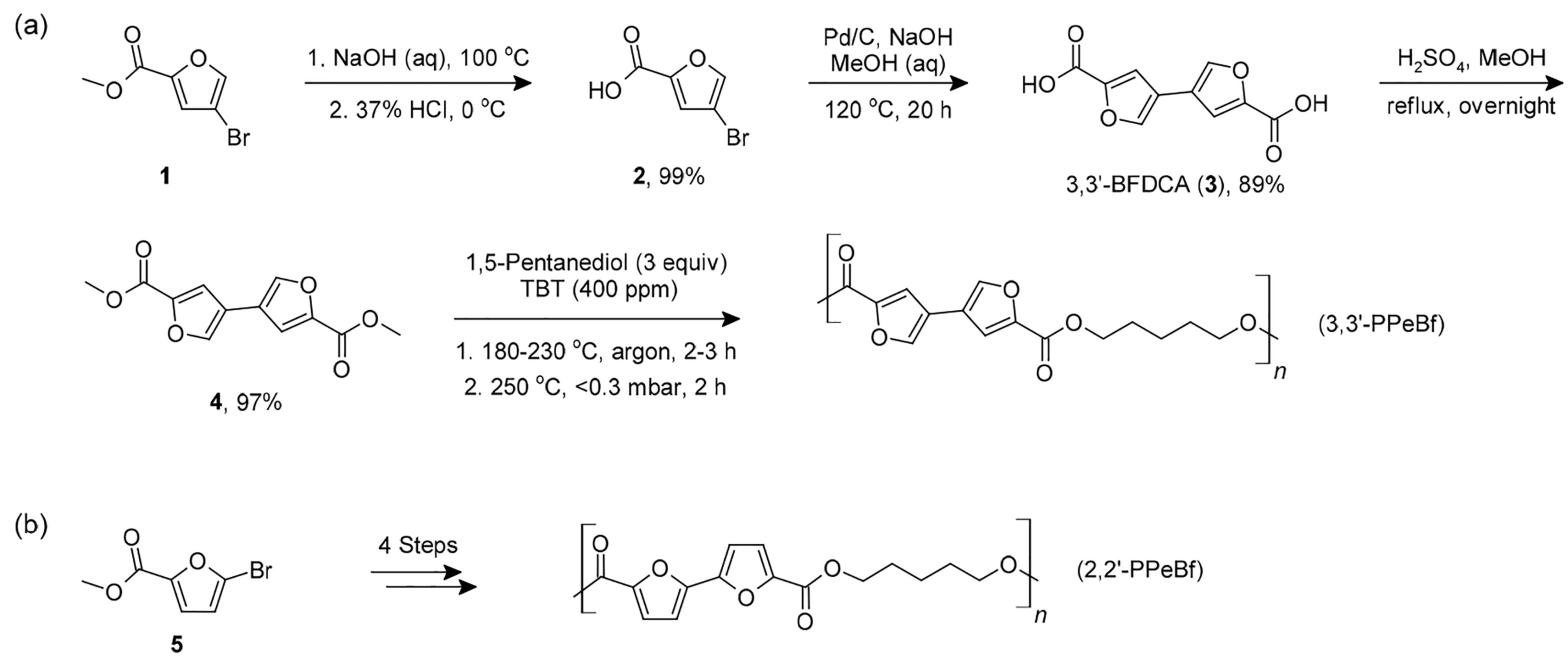
Synthesis Route to (a) 3,3′-BFDCA and 3,3′-PPeBf and (b) 2,2′-PPeBf

According to thermal analysis using DSC (Figure 1a, Table S1), 3,3′-PPeBf was semicrystalline, although crystallization was not observed during cooling from melt (Figure S17). In contrast, 2,2′-PPeBf appeared completely amorphous as evidenced by the lack of any notable endotherms or exotherms in DSC (Figure S18). Interestingly, 3,3′-bifuran units endowed 3,3′-PPeBf with a noticeably higher glass transition temperature compared to 2,2′-PPeBf (67 and 46 °C, respectively). Further DSC analysis revealed that the thermal properties of 3,3′-PPeBf were unstable: both the cold-crystallization exotherm and the melting endotherm became less prominent, and their peak temperatures changed after 10 weeks of storage under air (Figure 1a, Table S1). From dynamic mechanical analysis of melt-pressed films, decreased storage modulus *E*′ at higher temperatures (after the onset of cold crystallization) was observed in the air-exposed sample, due to more limited cold crystallization (Figure 1b). The peak of tan δ was found to be sensitive toward the process, as the peak position of tan δ shifted from 82 to 91 °C after a 10-week air-exposure period (Figure 1c). Additionally, the air-exposed samples were found to preserve their physical shape during thermal analysis despite temperatures high enough to melt 3,3′-PPeBf (*T*_m_ = 182 °C). 3,3′-PPeBf was also rendered insoluble in HFIP, although it was easily swelled by the solvent. The lack of dissolution however prevented further NMR study. The most conspicuous changes occurred in light transmittance of films, which based on UV–vis measurements approached a steady state after ca. 2–3 months (Figure 1d and 1e). The large red shift of the UV cutoff from 322 to 381 nm after aging meant major changes in conjugation must have happened. A small increase in transmittance was also observed at λ > 530 nm. When compared against 2,2′-PPeBf (Figure S19), it can be seen that 3,3′-PPeBf gains improved UV-blocking properties but only upon being exposed to air for a suitable period of time. The furan rings in the 3,3′-BFDCA unit are somewhat poorly conjugated, leading to narrower and weaker UV absorption initially. In terms of mechanical properties, the air aging mostly affected the elongation of 3,3′-PPeBF, which in tensile tests was found to go from ∼400% down to ∼70% after 1 month (Figure S20, Table S2). However, the polyester retained its high stiffness and strength relative to other similar polyesters (Table S2).^24^ These observations allowed us to conclude that 3,3′-PPeBf slowly reacts under air at ambient conditions, creating cross-links.

**Figure 1:**
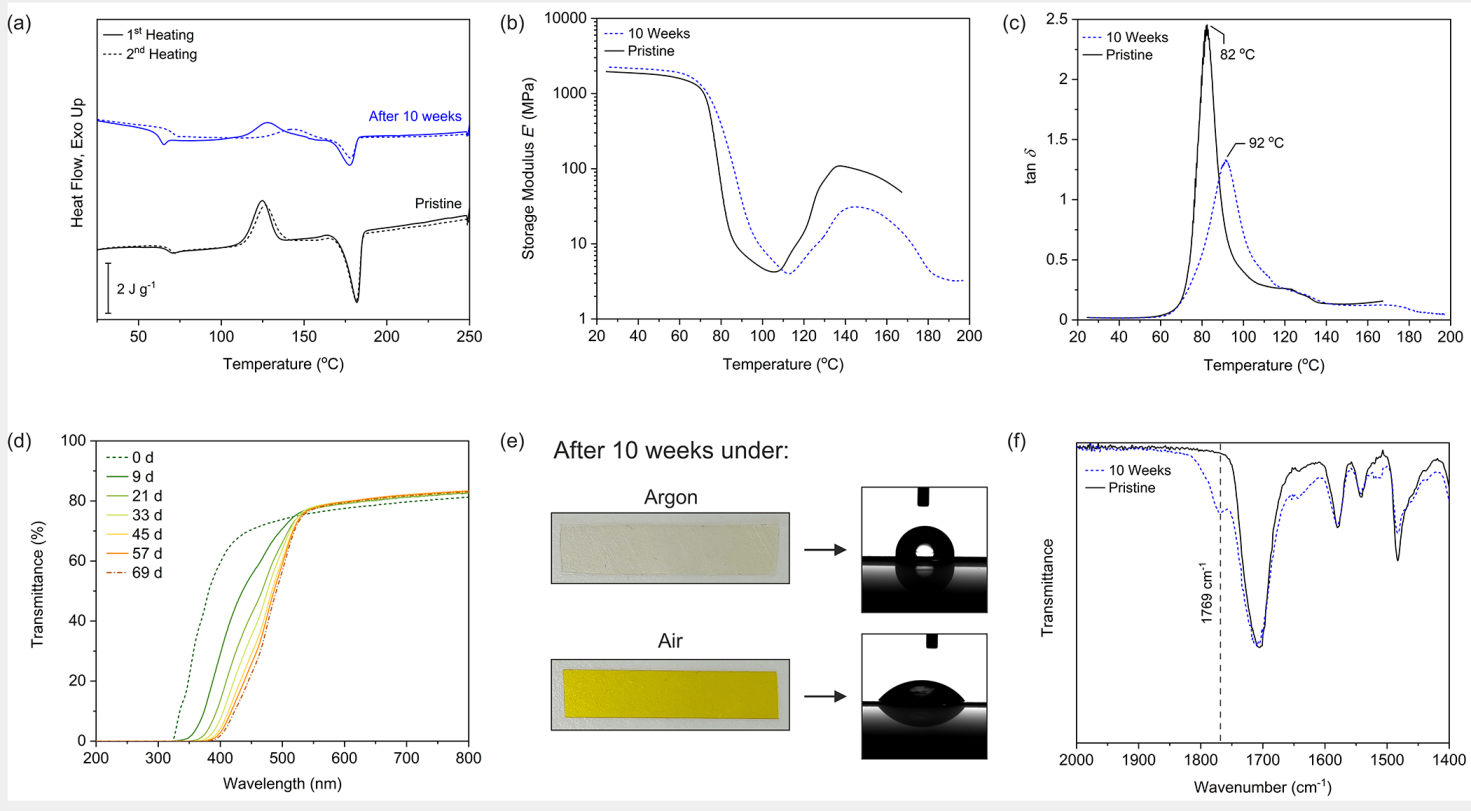
(a) DSC traces of pristine 3,3′-PPeBf films and samples stored under air for 10 weeks. (b) Storage modulus curves of pristine 3,3′-PPeBf films and samples stored under air for 10 weeks. (c) Tan δ curves of pristine 3,3′-PPeBf films and samples stored under air for 10 weeks. (d) The UV–vis transmittance of a 3,3′-PPeBf film specimen at various times after exposure to air. (e) Digital images illustrating the difference in the appearance and water contact angle of 3,3′-PPeBf film specimens stored under argon and air for 10 weeks. (f) ATR FTIR spectra of pristine films and films stored under air for 10 weeks.

To our great surprise, repeated measurements showed that the oxygen permeability of 3,3′-PPeBf was greatly influenced by prior exposure to air (Table 1, entries 5–9). In its initial state, 3,3′-PPeBf had O_2_ permeability ca. 3 times lower than the amorphous poly(ethylene terephthalate) (PET) sample used as a reference (BIF_PET_ = 3). With BIF_PET_ = 1.5, the oxygen permeability of 2,2′-PPeBf was intermediate between 3,3′-PPeBf and PET. Once exposed to air for at least 3 weeks, however, the oxygen permeability of 3,3′-PPeBf was found to have decreased by orders of magnitude. The O_2_ barrier performance of aged 3,3′-PPeBf is noteworthy, as it is comparable to ethylene-vinyl alcohol (EVOH) copolymers. Ethylene-vinyl alcohol copolymers with high vinyl alcohol content are top barrier materials against O_2_ transmission, but most of their properties are negatively affected by moisture. Permeation of oxygen can be increased by an order of magnitude or more in highly humid conditions, for instance.^25,26^ In contrast, 3,3′-PPeBf did not show sensitivity toward high levels of humidity during the permeability measurements (Table 1, entry 8). In terms of O_2_ barrier performance, the only reported furan polymers approaching this level of performance are poly(pentamethylene-2,5-furanoate) (PPeF) and 2,4-PBF. However, both are softer materials with glass transition temperatures close to room temperature (Tables S1 and S2), setting 3,3′-PPeBf apart with its high *T*_g_, semicrystalline character, and good mechanical properties.

**Table 1:** O_2_ Permeability (OP) of Prepared Film Specimens and Relevant Barrier Polymers from Previous Reports

Entry	Material Code	OP (mL μm m^–2^ d^–1^ atm^–1^)	BIF_PET_^g^	Conditions	Reference
1	PET	4638	1	23 °C, 0% RH	This study
2	PET	3630	1	23 °C, 0% RH	(27)
3	PET	1445	1	23 °C, 50% RH	(28)
4	2,2′-PPeBf	3080	1.5	23 °C, 0% RH	This study
5	3,3′-PPeBf^a^	1362	3	23 °C, 0% RH	This study
6	3,3′-PPeBf^b^	14.3	324	23 °C, 0% RH	This study
7	3,3′-PPeBf^c^	13.0	357	23 °C, 0% RH	This study
8	3,3′-PPeBf^d^	9.3	499	23 °C, 80% RH	This study
9	3,3′-PPeBf^e^	5.6	828	23 °C, 0% RH	This study
10	PPeF	16	227	23 °C, 0% RH	(27)
11	PPeF	5647	0.26	23 °C, 50% RH	(28)
12	PEF	702	5	23 °C, 0% RH	(27)
13	PEF	269	5.4	23 °C, 50% RH	(28)
14	2,4-PBF	22	nr^h^	23 °C, 0% RH	(22)
15	2,4-PBF	26	nr	23 °C, 85% RH	(22)
16	EVOH^f^	∼2	nr	20 °C, 0% RH	(25)
17	EVOH^f^	∼3	nr	20 °C, 50% RH	(25)
18	EVOH^f^	∼60	nr	20 °C, 90% RH	(25)

a Air exposure time measured: from freshly prepared film sample.

b Air exposure time measured: 3 weeks under dry ambient air.

c Air exposure time measured: 15 weeks.

d Air exposure time measured: 24 weeks.

e Air exposure time measured: 37 weeks.

f Ethylene-vinyl alcohol copolymer, 27% ethylene.

g BIF_PET_: barrier improvement factor vs PET sample.

h nr: not reported.

The way that these different polymers appear to achieve their oxygen barrier properties is worth a brief discussion. In the case of PPeF, the low permeability appears to be strongly linked to certain morphological features such as the presence of liquid-crystalline domains or mesophases within melt-pressed films.^27−29^ Only a single study has focused on 2,4-PBF so far, but the oxygen barrier effect has been supposed to arise due to special hydrogen bonding between the furan units. 3,3′-PPeBf, in contrast, appears to achieve its properties due to chemical changes under ambient conditions in air. This effectively converts it from a thermoplastic into a more complex cross-linked system. However, cross-linking is not known to dramatically alter the gas permeability of a polymer material.^30^ It is much more feasible that newly formed chemical structures, and their interactions, in the polymer act to hinder the permeation of oxygen: in ATR-FTIR spectra, broadening of the ester carbonyl peak at 1707 cm^–1^ with a shoulder appearing 1769 cm^–1^ was especially notable (Figure 1f). The wavenumber of the new shoulder peak appears consistent with the presence of lactones. Indeed, in air-aging experiments carried out on 3,3′-bifuran model compounds, namely, monomeric branched-chain dialkyl esters, two main reaction products were isolated, and both were assigned as unsaturated lactones based on NMR and mass analyses. Elemental analysis of 3,3′-PPeBf film samples also supported the insertion of oxygen (Figure S22). It should also be noted that the water contact angles of the films changed dramatically after air exposure, decreasing from 93.5° to 55.7° (Figure 1e, Table S3).

At this point, it is interesting to consider the apparent inertness of the monomer, dimethyl ester **4**, toward storage under ambient air. Cyclic voltammetry indicated that **4** is more difficult to oxidize to its radical cation than the corresponding 2,2′-bifuran (with measured oxidation potentials of 1.593 and 1.295 mV, respectively vs ferrocene). This contradicts the expectation that the more easily oxidized bifuran should give a polyester that is less stable under an oxidizing atmosphere.^31^ The stability difference between the monomer and the polymer could simply result from their different morphologies: the small-molecule monomer is a high-melting crystalline compound, which restricts gas molecule diffusion and mobility of the monomer molecules. In contrast, the polyester in its essentially amorphous form (as studied here) allows better mobility. The same should be true for the small-molecule model compounds in their melt state. This possible prerequisite is also satisfied by 2,2′-PPeBf, but we have found no signs of reaction under air at ambient conditions (after more than 6 months of storage). In spite of its more easily oxidized bifuran units, 2,2′-PPeBf appears to be as stable as the previously reported polyesters in the 2,2′-bifuran family, made with ethylene glycol or 1,4-butanediol, for example.^16,32^ Finally, we also note that both humidity and ambient light appeared to play roles in the air-aging processes of 3,3′-PPeBf. High humidity and the absence of incident light were found to result in lessened impact on properties or a slower process (see Supporting Information for details). It may also be possible that the polymerization catalyst(s) influences the process: for instance, it has been shown that the polymer preparation procedures used here are more than likely to result in the retention of the titanium catalyst within the polymer.^33^ Nevertheless, the 3,3′-BFDCA moieties must be ultimately responsible for the observed reactivity and changes under air, although at present the mechanism can be mostly speculated upon (see Supporting Information for further discussion).

Uncovering the mechanisms underlying the observed air-driven aging in 3,3′-PPeBf obviously merits further studies far beyond the scope of this communication. Based on current observations, the phenomenon is attributed to reactions taking place in the presence of O_2_, which appears to be required for the described changes to happen. As a result, multiple properties of the polyester were observed to undergo extensive modification. Considering the ease by which the process appears to be triggered and its benefits for achieving especially low O_2_ permeability, these results help broaden the scope of renewable high-performance furan-based materials.

## Supporting Information Available

Experimental procedures and additional figures and data, including ^1^H and ^13^C NMR, DSC, UV–vis, IR, and tensile testing results (PDF)
