# Supplementary material for: High Oxygen Barrier Polyester from 3,3′-Bifuran-5,5′-dicarboxylic Acid
Source: ACS Macro Lett. 2023 Jan 13;12(2):147–51. doi: 10.1021/acsmacrolett.2c00743 (PMC9948531; doi:10.1021/acsmacrolett.2c00743)
Supplement: Supplementary file 1 — mz2c00743_si_001.pdf [file mz2c00743_si_001.pdf]

SUPPORTING INFORMATION

High Oxygen Barrier Polyester from 3,3'-Bifuran-5,5'-Dicarboxylic Acid

Tuomo P. Kainulainen[a], Tomi A. O. Parviainen[a], Juho Antti Sirviö[b], Liam J. R. McGeachie[c], Juha P. Heiskanen[a*]

[a]*Research Unit of Sustainable Chemistry, University of Oulu, P.O. Box 4300, FI-90014 Oulu, Finland*

[b]*Fibre and Particle Engineering Research Unit, University of Oulu, P.O. Box 4300, FI-90014 Oulu, Finland*

[c]*Laboratory of Inorganic Chemistry, Environmental and Chemical Engineering, University of Oulu, P.O. Box 4300, FI-90014 Oulu, Finland*

[*]Email: juha.heiskanen@oulu.fi

Table of contents

Experimental procedures 3
Figure S1. ^{1}H NMR spectrum of 4-bromo-2-furoic acid (**2**) 9
Figure S2. ^{13}C NMR spectrum of 4-bromo-2-furoic acid (**2**) 9
Figure S3. ^{1}H NMR spectrum of 3,3'-bifuran-5,5'-dicarboxylic acid (**3**) 10
Figure S4. ^{13}C NMR spectrum of 3,3'-bifuran-5,5'-dicarboxylic acid (**3**) 10
Figure S5. ^{1}H NMR spectra of dimethyl 3,3'-bifuran-5,5'-dicarboxylate (**4**) 11
Figure S6. ^{13}C NMR spectrum of dimethyl 3,3'-bifuran-5,5'-dicarboxylate (**4**) 12
Figure S7. ^{1}H NMR spectrum of 3,3'-PPeBf 13
Figure S8. ^{1}H NMR spectrum of 2,2'-PPeBf 13
Figure S9. ^{1}H NMR spectrum of **9** 14
Figure S10. ^{13}C NMR spectrum of **9** 14
Figure S11. ^{1}H NMR spectrum of **10** 15
Figure S12. ^{13}C NMR spectrum of **10** 15
Figure S13. 2D NMR spectra of **10** 16
Figure S14. ^{1}H NMR spectrum of **11** 17
Figure S15. ^{13}C NMR spectrum of **11** 17
Figure S16. 2D NMR spectra of **11** 18
Figure S17. DSC traces from 1st and 2nd cooling of 3,3'-PPeBf 19
Figure S18. DSC heating and cooling traces for 2,2'-PPeBf 19
Figure S19. UV-vis transmittance curves of 3,3'-PPeBf and 2,2'-PPeBf films 20
Figure S20. Representative stress-strain curves for pristine and aged 3,3'-PPeBf films 20
Figure S21. ATR FTIR spectra for 3,3'-PPeBf samples 21
Figure S22. Comparison of elemental analysis results from 3,3'-PPeBf films 21
Table S1. Thermal properties of 3,3'-PPeBf and reported comparable polyesters 22
Table S2. Tensile properties of 3,3'-PPeBf and reported comparable polyesters 22
Table S3. Water contact angles measured in air 22
Figure S23. DMA curves from 4-week-old 3,3'-PPeBf film samples after storage at various conditions 23
Figure S24. UV-vis curves from 4-week-old 3,3'-PPeBF film samples after storage at various conditions 23
Table S4. Evaluation of aging factors for 3,3'-PPeBf films via UV-vis and DMA 24
References 27

Experimental procedures

General information

Aluminium TLC plates coated with silica gel 60 were used for TLC. Commercial solvents and chemicals were generally used without further purification unless otherwise specified. Commercial 10% Pd/C pre-wetted with water (50%) was used as a catalyst for coupling reactions. $CDCl_3$ (99.96% D) and $(CD_3)_2SO$ (99.80% D) were used as NMR solvents, both containing TMS (δ 0.00 ppm) as an internal reference. CF_3COOD (99.50% D) was used as a co-solvent for NMR analysis of polyesters.

Synthesis of precursors and polymers

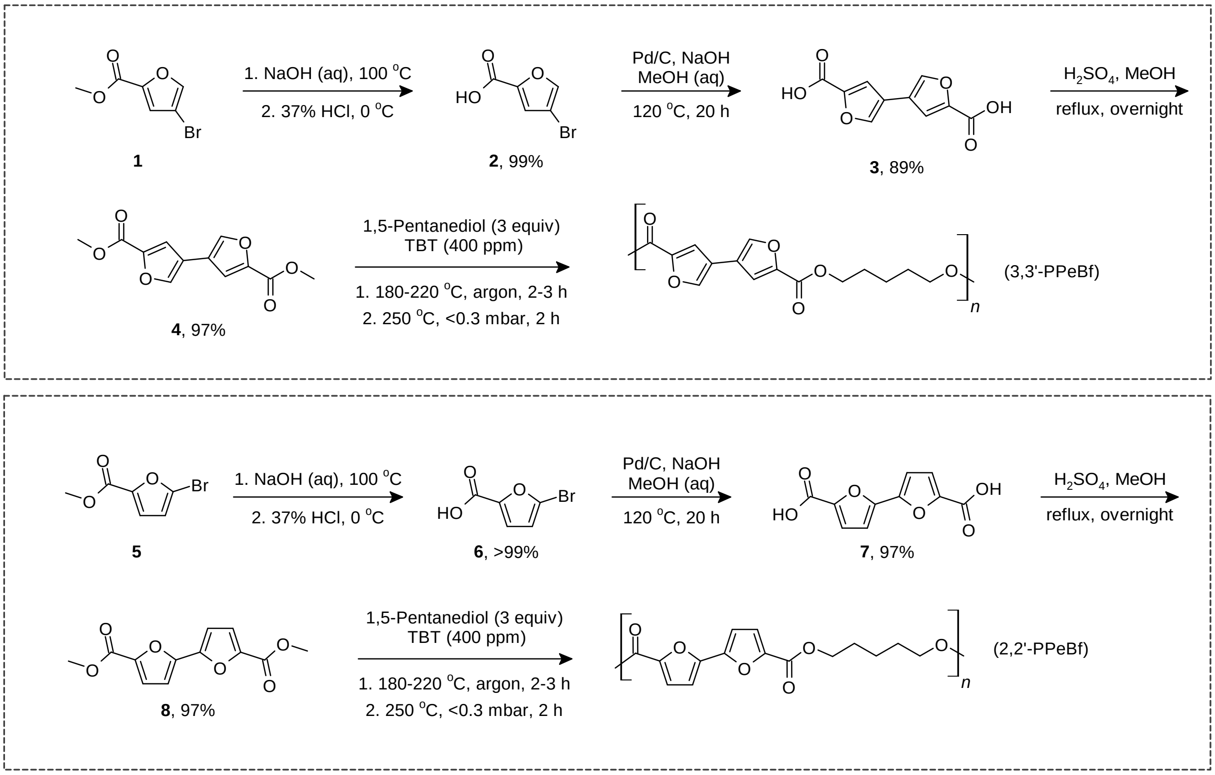

4-Bromofuran-2-carboxylic acid (2): Methyl 4-bromo-2-furoate (**1**; 10.07 g, 48.6 mmol) and NaOH (9.83 g, 5 equiv) were mixed in 100 mL of deionized water in a 250 mL round bottom flask equipped with a reflux condenser. The mixture was refluxed for 2 hours, after which the ester could no longer be detected using TLC (eluent: EtOAc/hexane 1:1). While cooling on an ice-bath, the reaction mixture was acidified (pH ~1) with 37% hydrochloric acid, and the product was extracted into ethyl acetate. The combined organic phases were dried over anhydrous Na_2SO_4, filtered, and evaporated to dryness. The product, **2**, was collected as a white solid in 99% yield (9.17 g). ^1H NMR (400 MHz, $(CD_3)_2SO$, ppm): δ 13.46 (br s, 1H), 8.17 (d, J = 0.9 Hz, 1H), 7.40 (d, J = 0.9 Hz, 1H). $^{13}C\{^1H\}$ NMR (100 MHz, $(CD_3)_2SO$, ppm): δ 158.4, 145.6, 145.4, 119.6, 100.5.

3,3'-Bifuran-5,5'-dicarboxylic acid (3): Compound **2** (4.00 g, 20.9 mmol), NaOH (2.13 g, 2.5 equiv), and the Pd/C catalyst (445.4 mg, 0.01 equiv Pd) were added together to a mixture of distilled water (112 mL) and

methanol (8 mL) in a round-bottom pressure flask. The headspace was flushed with argon, and the flask was sealed with a threaded PTFE cap. The mixture was vigorously stirred at 120 °C for up to 24 hours, after which the starting material could no longer be detected by TLC. The solid Pd/C catalyst was filtered off and rinsed with deionized water. The filtrate was acidified with 37% hydrochloric acid, to pH = ~1, resulting in a light-colored precipitate. The precipitate was collected via filtration to a Büchner funnel and washed with deionized water. After drying at 40 °C overnight, **3** was collected as an off-white solid (2.07 g, 89%). ^{1}H NMR (400 MHz, $(CD_3)_2SO$, ppm): δ 13.24 (br s, 2H), 8.32 (d, J = 0.9 Hz, 2H), 7.62 (d, J = 0.9 Hz, 2H). ^{13}C{^{1}H} NMR (100 MHz, $(CD_3)_2SO$, ppm): δ 159.2, 145.6, 143.2, 118.4, 116.0.

*Dimethyl-3,3'-bifuran-5,5'-dicarboxylate (**4**)*: Dicarboxylic acid **3** (3.37 g, 15.2 mmol) was added to a 500 mL round bottom flask, along with 340 mL of methanol and 3.3 mL of 97% sulfuric acid. The mixture was refluxed under vigorous magnetic stirring. After 24 hours the starting material could no longer be detected (TLC), and the mixture was allowed to cool to room temperature. The solution was concentrated with a rotary evaporator, to about one-quarter volume, and diluted back with deionized water. The precipitate was filtered using a Büchner funnel and washed with deionized water, affording **4** as an off-white powder (3.69 g, 97%). For use in polymerizations, the product was purified further by passing it through a layer of silica in warm chloroform. ^{1}H NMR (400 MHz, $CDCl_3$, ppm): δ 7.74 (d, J = 0.8 Hz, 2H), 7.29 (d, J = 0.8 Hz, 2H), 3.93 (s, 6H). ^{1}H NMR (400 MHz, $(CD_3)_2SO$, ppm): δ 8.40 (d, J = 0.9 Hz, 2H), 7.77 (d, J = 0.9 Hz, 2H), 3.83 (s, 6H). ^{13}C{^{1}H} NMR (100 MHz, $(CD_3)_2SO$, ppm): δ 158.2, 144.4, 143.9, 118.4, 116.6, 51.9. T_m (DSC): 235 °C.

*5-Bromofuran-2-carboxylic acid (**6**)*: The title compound was synthesized from commercially available methyl 5-bromo-2-furoate (**5**, 10.17 g, 49.1 mmol) by following the synthetic procedure for **2**. Yield: >99% (9.41 g). ^{1}H NMR (400 MHz, $(CD_3)_2SO$, ppm): δ 13.36 (br s, 1H), 7.25 (d, J = 3.4 Hz, 1H), 6.80 (d, J = 3.4 Hz, 1H).

*2,2'-Bifuran-5,5'-dicarboxylic acid (**7**)*: The title compound was prepared from **6** by following the procedure described for dicarboxylic acid **3**. Yield: 2.25 g (97%). ^{1}H NMR (400 MHz, $(CD_3)_2SO$, ppm): δ 13.36 (br s, 2H), 7.35 (d, J = 3.7 Hz, 2H), 7.08 (d, J = 3.7 Hz, 2H).

*Dimethyl-2,2'-bifuran-5,5'-dicarboxylate (**8**)*: The title compound was prepared from dicarboxylic acid **7** (2.23 g, 10.0 mmol) following the esterification used to prepare **4**. The product was a white powder (2.45 g, 97%). It was further purified as described for **4**. ^{1}H NMR (400 MHz, $CDCl_3$, ppm): δ 7.25 (d, J = 3.7 Hz, 2H), 6.90 (d, J = 3.7 Hz, 2H), 3.92 (s, 6H).

Poly(pentamethylene-3,3'-bifuranoate) (3,3'-PPeBf): 1,5-pentanediol (2.6 mL, 3 equiv) and the catalyst solution (15 mM tetrabutyl titanate in dry toluene, 400 ppm tetrabutyl titanate relative to the bifuran monomer) were first briefly mixed in a dry 50 mL round-bottom flask under argon with a small PTFE-coated stirring magnet. Afterwards, **4** (2.00 g, 8 mmol) was added in. The reaction flask was subsequently connected to a short-path distillation bridge together with vacuum and argon lines. The system was evacuated to 10 mbar and then filled with argon (99.995%) for at least five times. To initiate the transesterification, the flask was first rapidly heated to 230 °C for 15 min and then maintained at 180 °C. After methanol ceased to distill over (90–

120 min from the start of the transesterification), pressure inside the system was decreased in gradual manner to <10 mbar over 60–75 min with distillation of excess diol. After this, the polycondensation was initiated by further decrease of pressure to 0.1–0.3 mbar and an increase in temperature to 250 °C. These conditions were maintained for 2 h under slow magnetic stirring. After the system had cooled, 1,1,1,3,3,3-hexafluoroisopropanol (HFIP) was added into the flask under argon to dissolve the product. The solution (ca. 10% w/v) was then allowed to drain through a funnel with a fritted glass disc (porosity: 1). The flask and the funnel were rinsed 3 times with few-milliliter batches of HFIP. The filtered solution was mixed with methanol (300 mL) to precipitate the polymer as a white fibrous aggregate, which was filtered off and vacuum dried at 50–60 °C to a constant mass. Typical yield assuming infinite degree of polymerization: 93% (2.18 g). ^{1}H NMR (400 MHz, $CDCl_3/CF_3COOD$ 2:1 v/v, ppm): δ 7.82 (s, 2H), 7.43 (s, 2H), 4.46 (t, J = 6.6 Hz, 4H), 1.92 (quin, J = 6.8 Hz, 4H), 1.63 (m, 2H). Anal. Calcd for $C_{15}H_{14}O_6$: C, 62.07; H, 4.86; O, 33.07. Found: C, 61.66; H, 4.84; O, 30.80.

Poly(pentamethylene-2,2'-bifuranoate) (2,2'-PPeBf): 2,2'-PPeBf was prepared in manner analogous to 3,3'-PPeBf by using diester **8** (2.00 g, 8 mmol). The resultant polyester was dissolved in ca. 20 mL of HFIP and chloroform (3:1 v/v). After filtration, precipitation, and drying, the polymer was collected as a dense white clump. Yield: 83% (1.93 g). ^{1}H NMR (400 MHz, $CDCl_3/CF_3COOD$ 2:1 v/v, ppm): δ 7.37 (d, J = 3.7 Hz, 2H), 6.93 (d, J = 3.7 Hz, 2H), 4.47 (t, J = 6.5 Hz, 4H), 1.92 (m, 4H), 1.63 (m, 2H).

Model compound synthesis and air-aging experiments

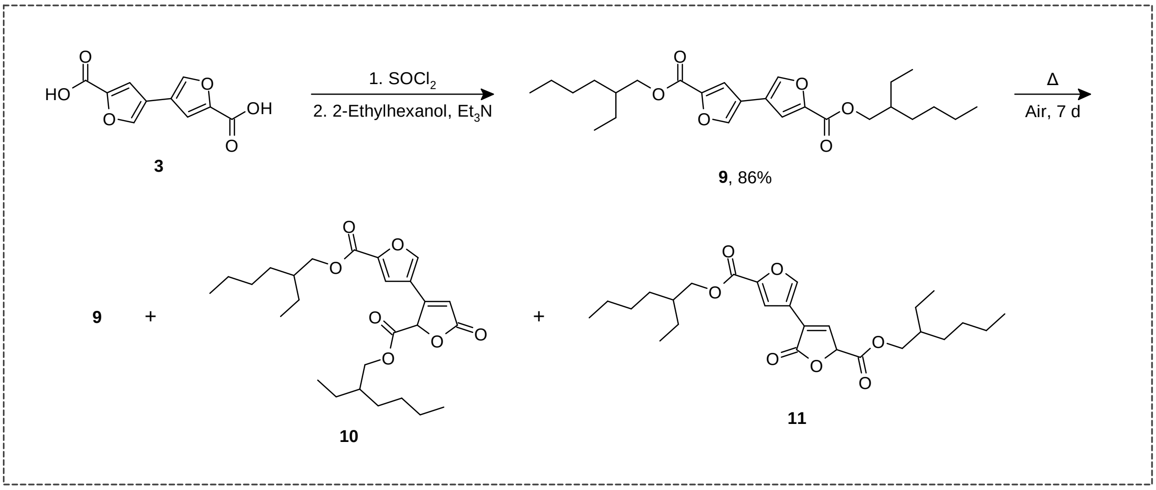

*Di(2-ethylhexyl)-3,3'-bifuran-5,5'-dicarboxylate (**9**)*: Dicarboxylic acid **3** (0.89 g, 4.0 mmol), crushed into fine powder, was reacted with $SOCl_2$ (0.9 mL, 3 equiv) and few drops of *N*,*N*-dimethylformamide in refluxing dichloromethane (10 mL) for 12 h. For the following step, the volatiles were evaporated under reduced pressure. The dried crude diacid chloride was resuspended into dry dichloromethane (40 mL) at 0 °C, to which triethylamine (1.4 mL, 2.5 equiv) was added dropwise. Then, 2-ethylhexanol (1.6 mL, 2.5 equiv) was introduced into the mixture dropwise over 15 min. The mixture was allowed to return to room temperature, with the reaction proceeding for 1 h. After being successively washed with 20 mL saturated $NaHCO_3$ solution,

2x20 mL deionized water, and 20 mL brine, the dichloromethane solution was passed through a short silica plug and evaporated dry under vacuum. The product was further purified by successive filtrations though silica. The product was an off-white solid (1.54 g, 86%). ^{1}H NMR (400 MHz, $CDCl_3$, ppm): δ 7.74 (d, J = 0.8 Hz, 2H), 7.25 (d, J = 0.8 Hz, 2H), 4.29–4.20 (m, 4H), 1.71 (sept, J = 6.1 Hz, 2H), 1.48–1.30 (m, 16H), 0.97–0.88 (m, 12H). $^{13}C\{^{1}H\}$ NMR (100 MHz, $CDCl_3$, ppm): δ 158.6, 145.7, 141.9, 118.6, 115.9, 67.6, 38.8, 30.7, 28.9, 23.8, 22.9, 14.0, 11.0.

Model compound aging experiment: Diester **9** (0.5 g) was first heated to 100 °C in 10 mL round-bottom flask to maintain it in a molten state. The melt was mixed under air for 7 days using a small PTFE-coated stirring bar. The evolution of the mixture was followed via TLC, which showed new spots appearing after just 1 d. The resulting mixture was fractionated via column chromatography (silica, ethyl acetate/hexane 1:3) into three initial fractions. The unreacted bifuran (**9**) eluted first (210.9 mg), followed by a roughly 6:4 molar mixture of two new compounds **10** and **11** (96.3 mg), and finally a fraction composed of **11** (46.8 mg). The mixture of compounds **10** and **11** was fractionated again to obtain a smaller sample of **10** (26.0 mg). Both compounds were pale yellow viscous oils. **10**: ^{1}H NMR (400 MHz, $CDCl_3$, ppm): δ 8.05 (s, 1H), 7.32 (d, J = 0.7 Hz, 1H), 6.28 (d, J = 1.7 Hz, 1H), 5.67 (d, J = 1.7 Hz, 1H), 4.29–4.20 (m, 2H), 4.17–4.07 (m, 2H), 1.71 (sept, J = 6.1 Hz, 1H), 1.60–1.54 (m, 1H), 1.46–1.26 (m, 16H), 0.96–0.91 (m, 6H), 0.87–0.81 (m, 6H). $^{13}C\{^{1}H\}$ NMR (100 MHz, $CDCl_3$, ppm): δ 171.3, 166.3, 158.0, 152.9, 146.6, 146.1, 117.9, 115.1, 114.6, 79.7, 69.3, 68.0, 38.8, 38.6, 30.3, 30.1, 28.9, 28.7, 23.7, 23.5, 22.9, 22.8, 14.0, 13.9, 10.9, 10.8. HRMS: (m/z) calculated for $C_{26}H_{38}O_7$ $[M+H]^+$: 463.26903, found 463.27065. **11**: ^{1}H NMR (400 MHz, $CDCl_3$, ppm): δ 8.34 (s, 1H), 7.40 (d, J = 2.2 Hz, 1H), 7.35 (d, J = 0.8 Hz, 1H), 5.57 (d, J = 2.2 Hz, 1H), 4.29–4.20 (m, 2H), 4.17–4.07 (m, 2H), 1.74–1.61 (m, 2H), 1.46–1.28 (m, 16H), 0.96–0.88 (m, 12H). $^{13}C\{^{1}H\}$ NMR (100 MHz, $CDCl_3$, ppm): δ 170.1, 165.5, 158.4, 145.9, 145.7, 139.4, 125.3, 116.4, 115.1, 78.8, 69.2, 67.8, 38.8, 38.6, 30.3, 30.2, 28.9, 28.8, 23.7, 23.6, 22.9, 22.8, 13.99, 13.95, 10.94, 10.88. HRMS: (m/z) calculated for $C_{26}H_{38}O_7$ $[M+H]^+$: 463.26903, found 463.26884.

Sample processing and analysis

Solution viscometry: Flow times of dilute polymer solutions (c = 0.5 g/dL) were determined in a 60:40 w/w mixture of phenol and 1,1,2,2-tetrachloroethane using a micro-Ubbelohde viscometer. To first dissolve the polymers, the solutions were gently heated (50–60 °C) under argon. Once dissolved, the viscometer was filled with the solution to be measured and allowed to equilibrate for 30 min. Measurements were performed with the viscometer submerged in a water bath set to maintain a temperature of 30.0 °C. The flow times were timed by hand with five repeat measurements, from which an average was calculated. Relative viscosities were calculated according to η_{rel},= t/t_0 where t is the flow time of the polymer solution and t_0 is the flow time of the pure solvent. From this value of η_{rel}, intrinsic viscosities $[\eta]$ were estimated using the Billmeyer relation,[1] $[\eta] = 0.25 \times [(\eta_{rel} - 1 + 3 \times \ln \eta_{rel})/c]$, where c is the concentration (g/dL) of the polymer solution.

Melt-pressing: Carefully dried polymer samples were melt-pressed into films using a heated hydraulic press (Fontijne LabEcon 300). The aluminium press plates (thickness 3 mm) were covered with polytetrafluoroethylene (PTFE) coated glass-fiber mats. The press, with plates pre-heated to 200 °C, was first used to melt the samples without applying compression. After 3 min of melt time, the press was closed with a force of 20 kN (held for 1 min), which was then increased to 40 kN (held for 1 min). The sample was cooled to 30–40 °C using the integrated water circulation, and the films were peeled from the PTFE-coated mat once cooled. Film thickness, controlled by a glass-fiber frame, was 100–200 μm. 3,3'-PPeBf samples were generally stored under argon, covered from light, unless otherwise necessitated by experiments.

Differential scanning calorimetry (DSC): Small pieces cut from melt-pressed films, ca. 4 mg, were placed in 45 μL Al pans that were then sealed with pierced lids. Samples were heated (Mettler DSC821e) at a rate of 10 °C/min from 20 to 250 °C and held at 250 °C for 3 min before reversing. Measurements were carried out under N_2 (99.995%), with a gas flow rate of 50 mL/min.

Oxygen permeability measurement: Oxygen permeabilities were measured (Mocon OxTran 2/20) from samples cut from melt-pressed films (test area: 5 cm^2). Measurements were done under various conditions of relative humidity at 23 °C.

Dynamic mechanical analysis (DMA): Melt-pressed film pieces were analyzed in tensile mode (DMA Q800) over the temperature range of 20–200 °C, at a heating rate of 3 °C/min, at 1 Hz and 0.08% strain with 125% force track. Sample dimensions were ca. 20×5×0.1 mm^3.

Tensile testing: Tensile tests (Instron 5544) were conducted under conditions of controlled temperature and humidity (23 °C and 50% RH), and the samples were stored under the same conditions for at least 48 h prior to testing. A minimum of 5 samples were evaluated. Samples were rectangular pieces with width of 5 mm. Gage length was 30 mm and cross-head speed 5 mm/min.

Attenuated total reflectance FTIR spectroscopy (ATR FTIR): ATR FTIR spectra were acquired using Perkin Elmer Spectrum One. 16 scans were acquired at a resolution of 2 cm^{-1}.

Water contact angle: The contact angle measurements were conducted using Krüss DSA25 (Germany) Drop Shape Analyzer with high-speed camera and drop analyzing software. During the measurement, a 5 μL droplet of water was added on the top of the film and the contact angle was recorded after 10 s. For each sample, five droplets on different locations were analyzed and the results are presented as average.

Cyclic voltammetry: Cyclic voltammetry (CV) was performed with an Gamry reference 600 Potentiostat and analyzed with the Gamry Echem Analyst software (version 6.33). CV was carried out in a five-neck electrochemical cell. A 3.0 mm diameter glassy carbon (GC) electrode was used as the working electrode. A platinum wire was used as an auxiliary electrode. The reference electrode was Ag/Ag^+ (0.1 M in acetonitrile). Prior to use, the GC electrode was sanded and polished with alumina slurry on a micro-cloth. The electrode was then rinsed with an ethanol, deionized water and acetonitrile. Monomer (5 mM) was dissolved in

supporting electrolyte consisting of *tetra-n*-butylammonium tetrafluoroborate ($TBABF_4$) thrice recrystallized from absolute ethanol with distilled acetonitrile as solvent. Scan rate of 100 mV/s was used. All experiments were conducted under argon atmosphere.

Aging experiments: To observe the aging process of the 3,3'-PPeBf films under various combinations of potential aging factors (air, humidity, and ambient indoor lighting), samples were prepared from pristine films immediately after melt-pressing. These samples were stored in sealed glass desiccators under the following conditions for 4 weeks, divided into eight sets of film strips:

Experiment code	Atmosphere	RH (%)	Light exposure
A	Air	<10	No
B	Air	>80	No
C	Air	<10	Yes
D	Air	>80	Yes
E	Argon	<10	No
F	Argon	>80	No
G	Argon	<10	Yes
H	Argon	>80	Yes

Samples stored under "<10% RH" conditions were stored over dry silica gel granules, whereas conditions of high humidity (80–90%) were created using a cup of water containing a large sediment of Na_2CO_3. Sample desiccators containing an argon atmosphere were first held under reduced pressure (<20 mbar) for degassing and then backfilled five times with 99.999% argon. Dark samples were kept shielded from any light inside a sealed metal cabinet.

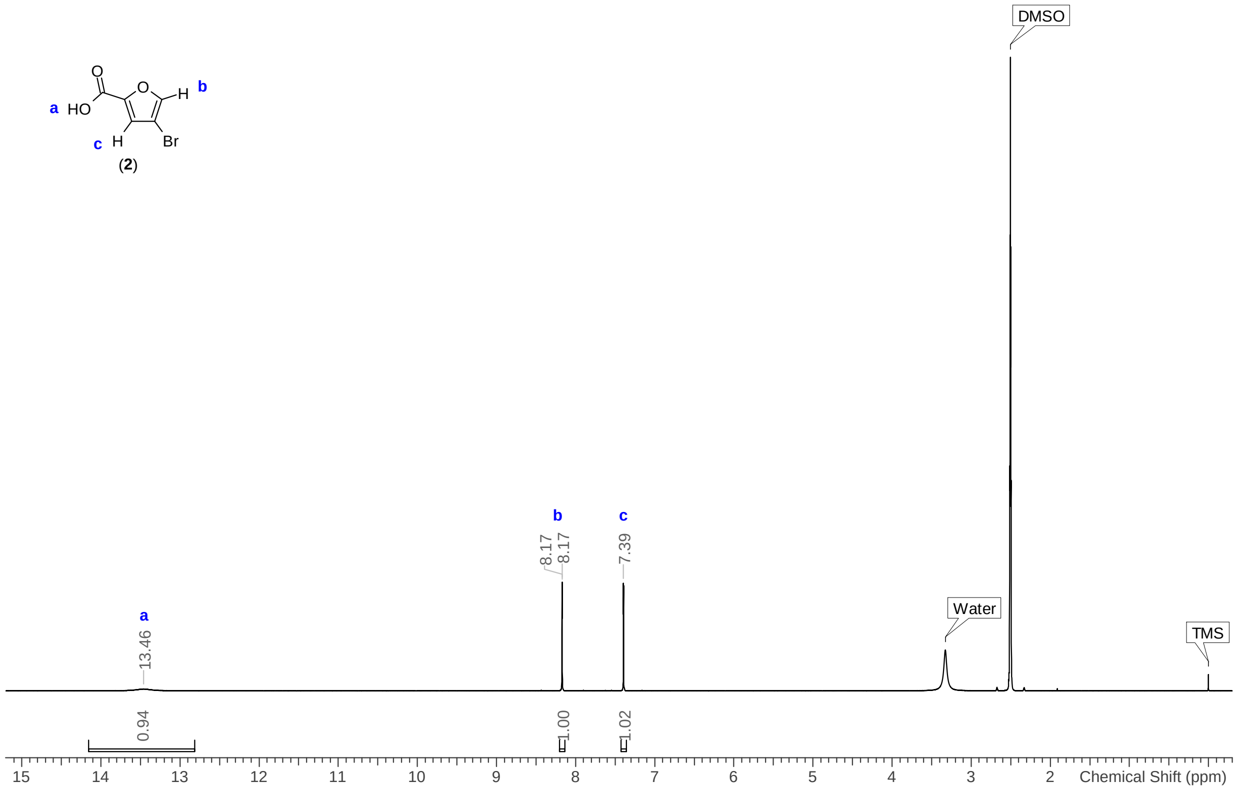

Figure S1. ^{1}H NMR spectrum of 4-bromo-2-furoic acid (**2**) in $(CD_3)_2SO$.

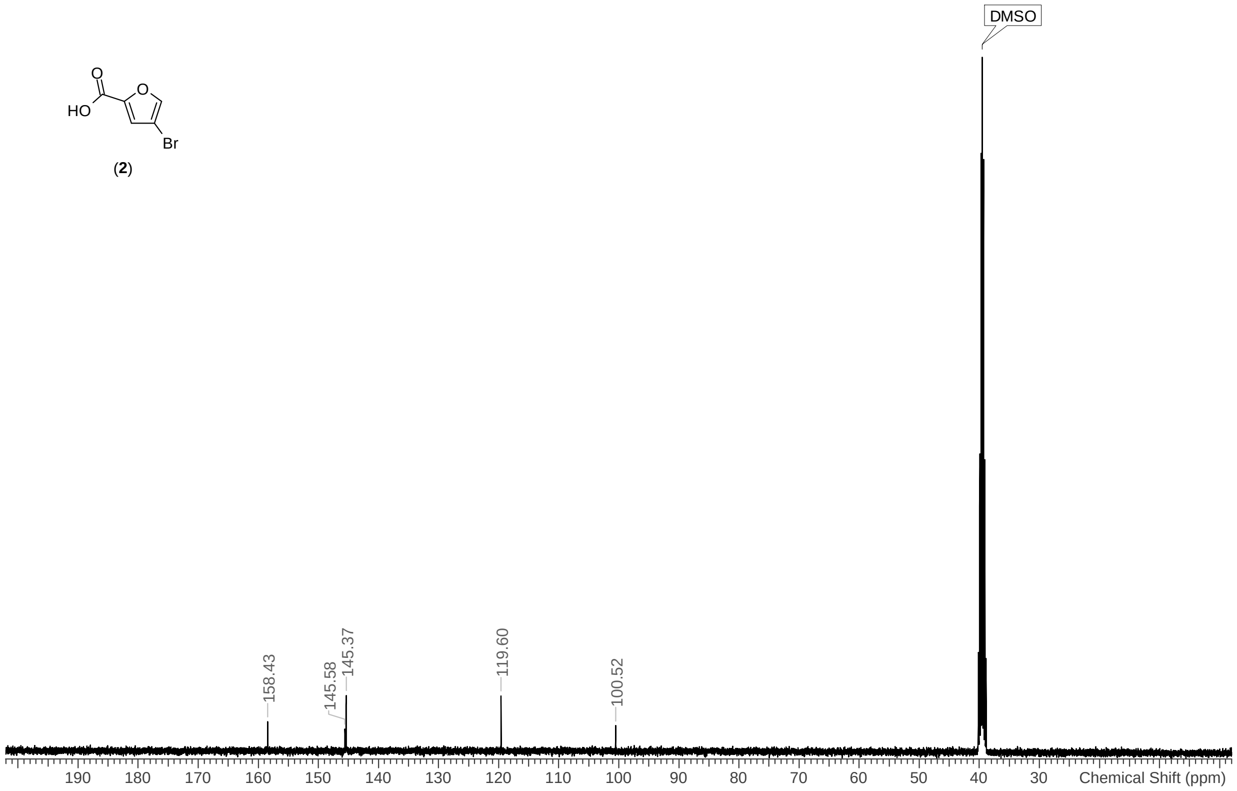

Figure S2. ^{13}C NMR spectrum of 4-bromo-2-furoic acid (**2**) in $(CD_3)_2SO$.

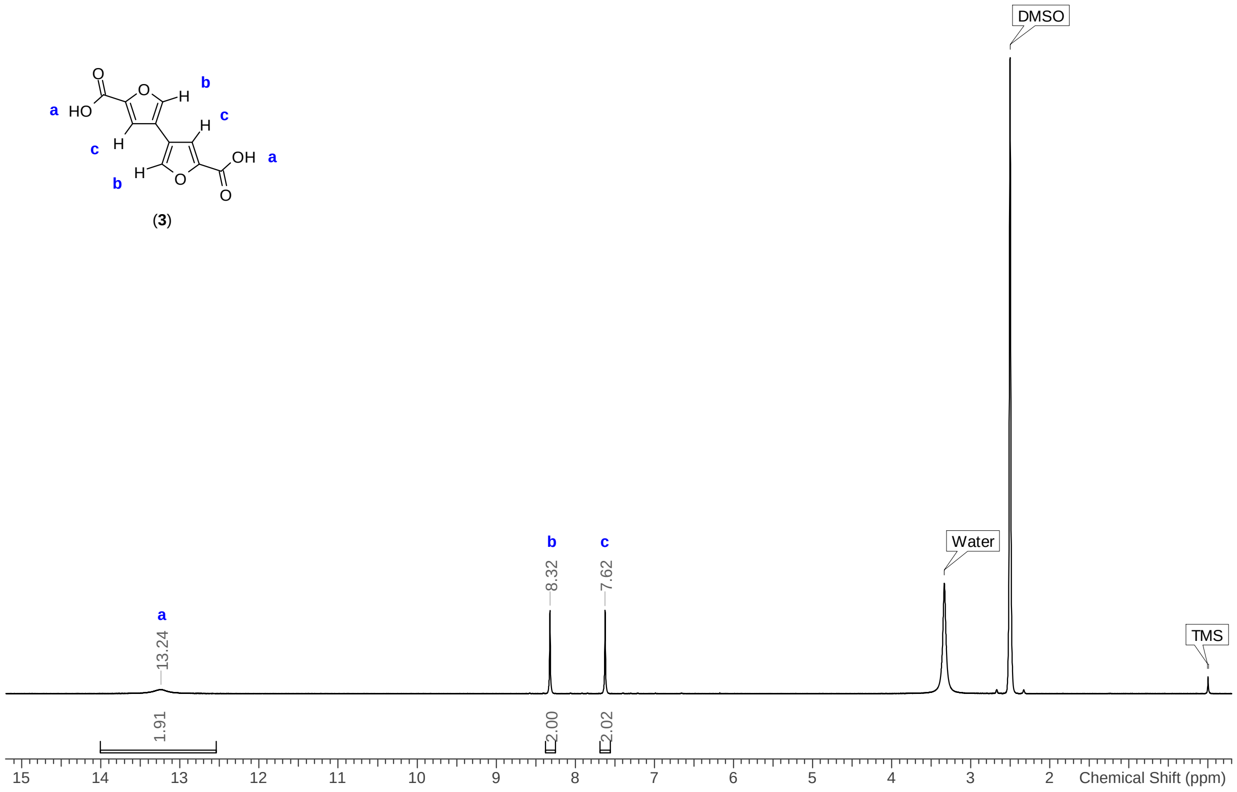

Figure S3. ^{1}H NMR spectrum of 3,3'-bifuran-5,5'-dicarboxylic acid (**3**) in $(CD_3)_2SO$.

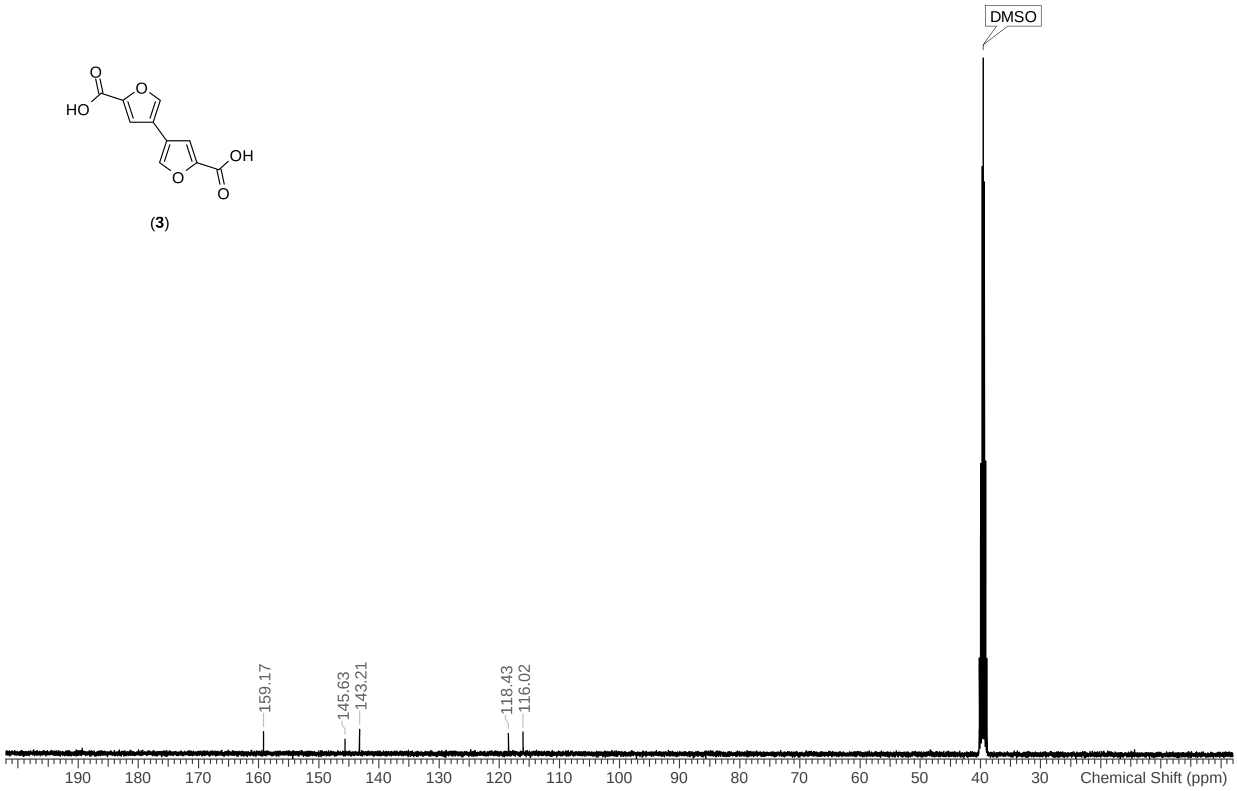

Figure S4. ^{13}C NMR spectrum of 3,3'-bifuran-5,5'-dicarboxylic acid (**3**) in $(CD_3)_2SO$.

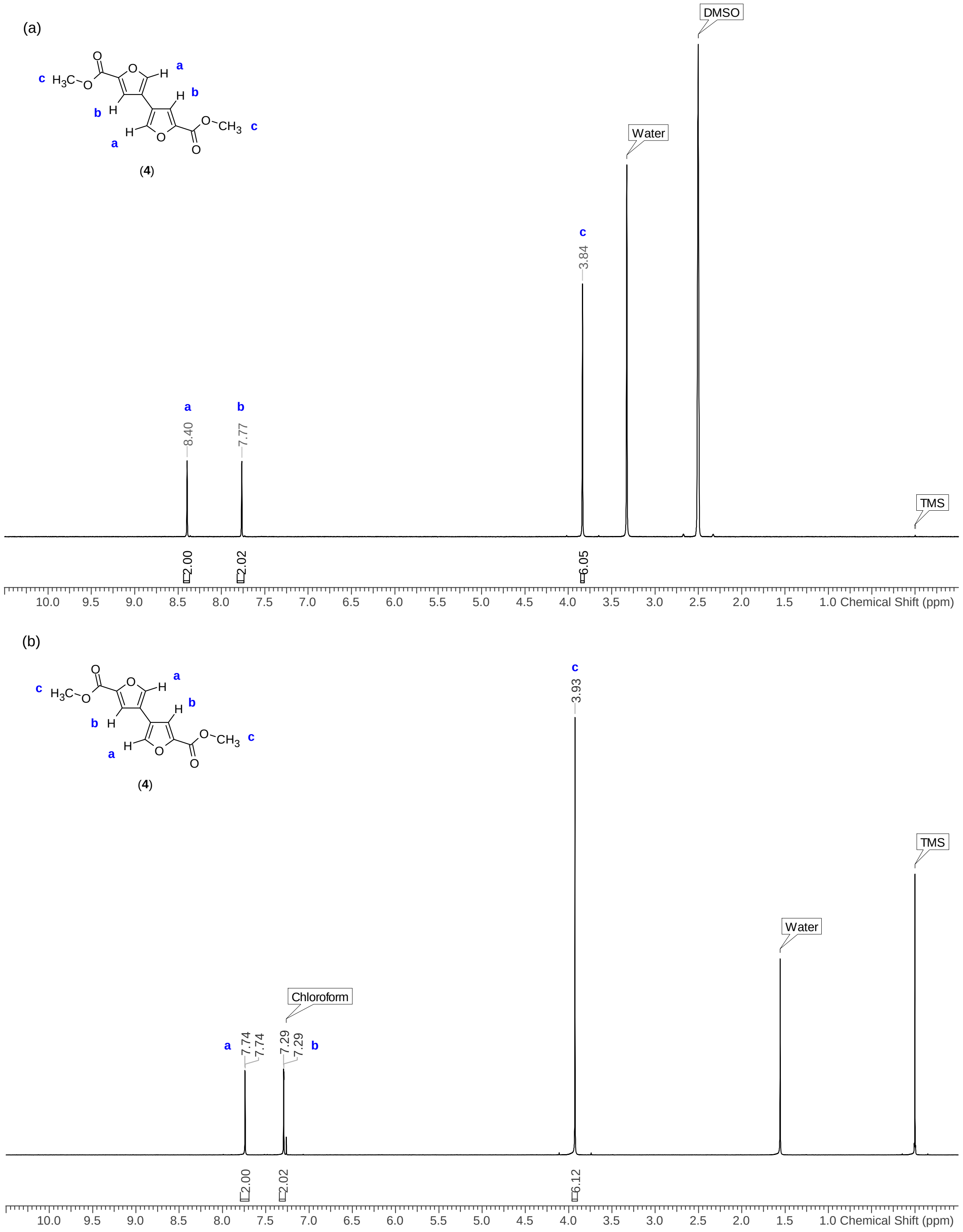

Figure S5. ^{1}H NMR spectra of dimethyl 3,3’-bifuran-5,5’-dicarboxylate (**4**) in (a) $(CD_3)_2SO$ (b) $CDCl_3$.

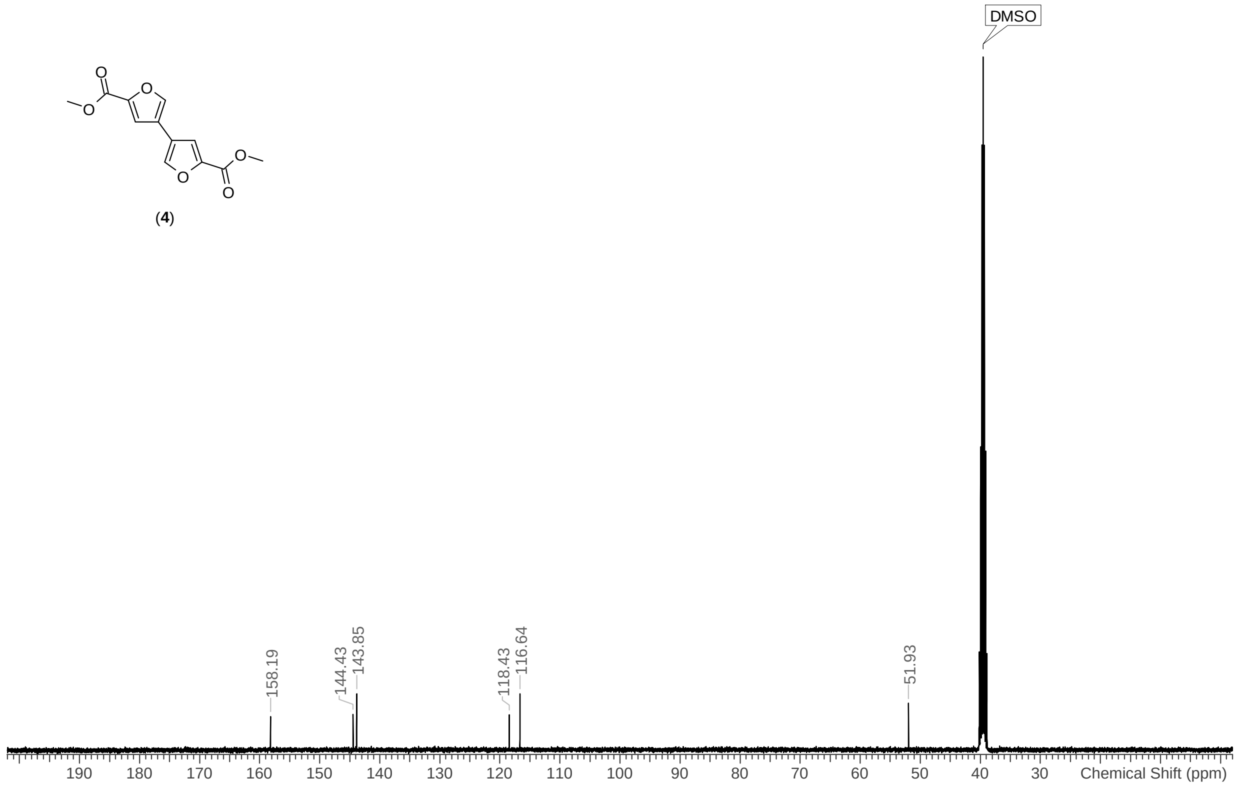

Figure S6. ^{13}C NMR spectrum of dimethyl 3,3'-bifuran-5,5'-dicarboxylate (**4**) in $(CD_3)_2SO$.

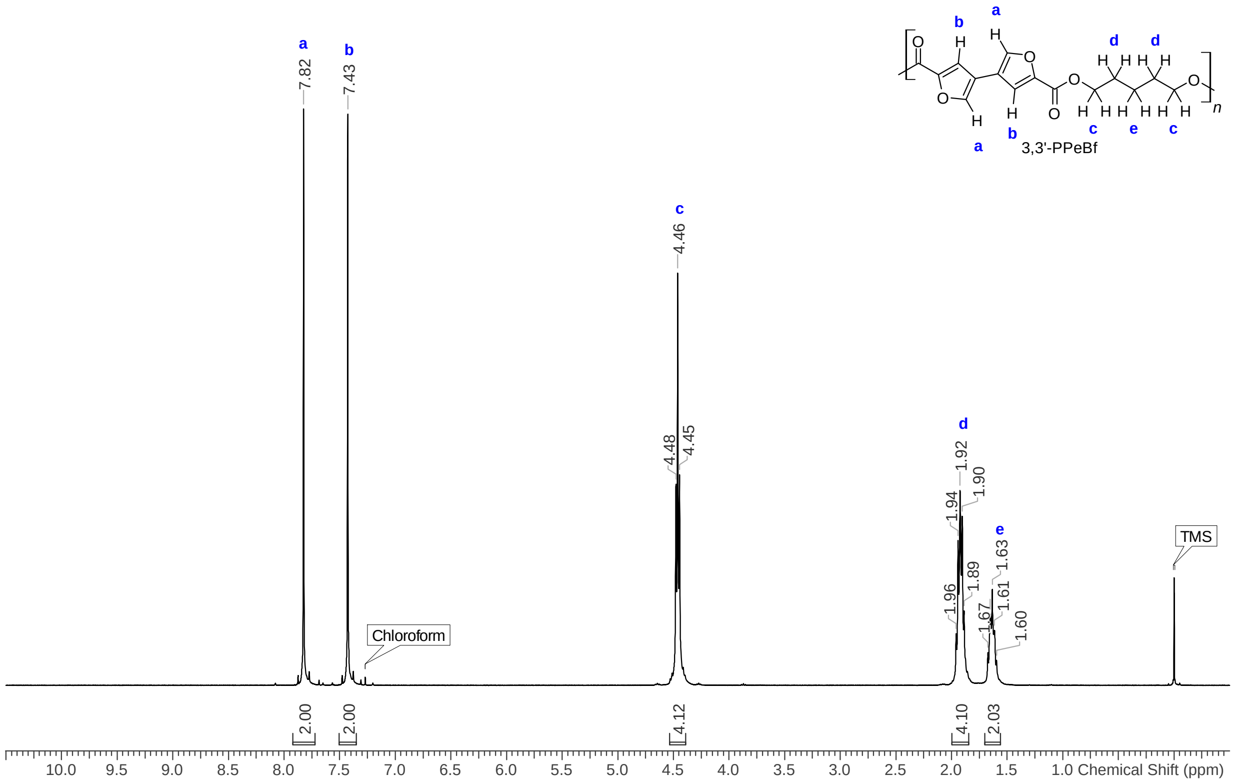

Figure S7. ^{1}H NMR spectrum of 3,3'-PPeBf in $CDCl_3/CF_3COOD$, 2:1 v/v.

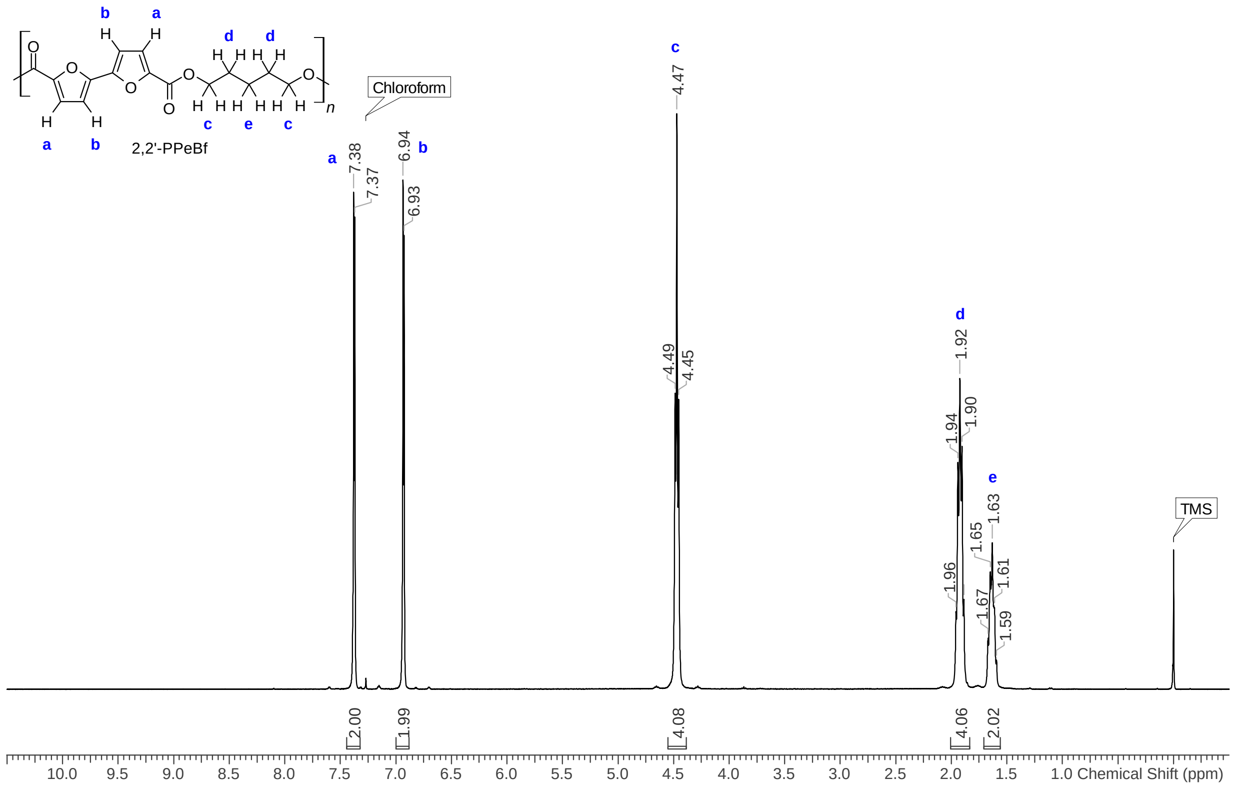

Figure S8. ^{1}H NMR spectrum of 2,2'-PPeBf in $CDCl_3/CF_3COOD$, 2:1 v/v.

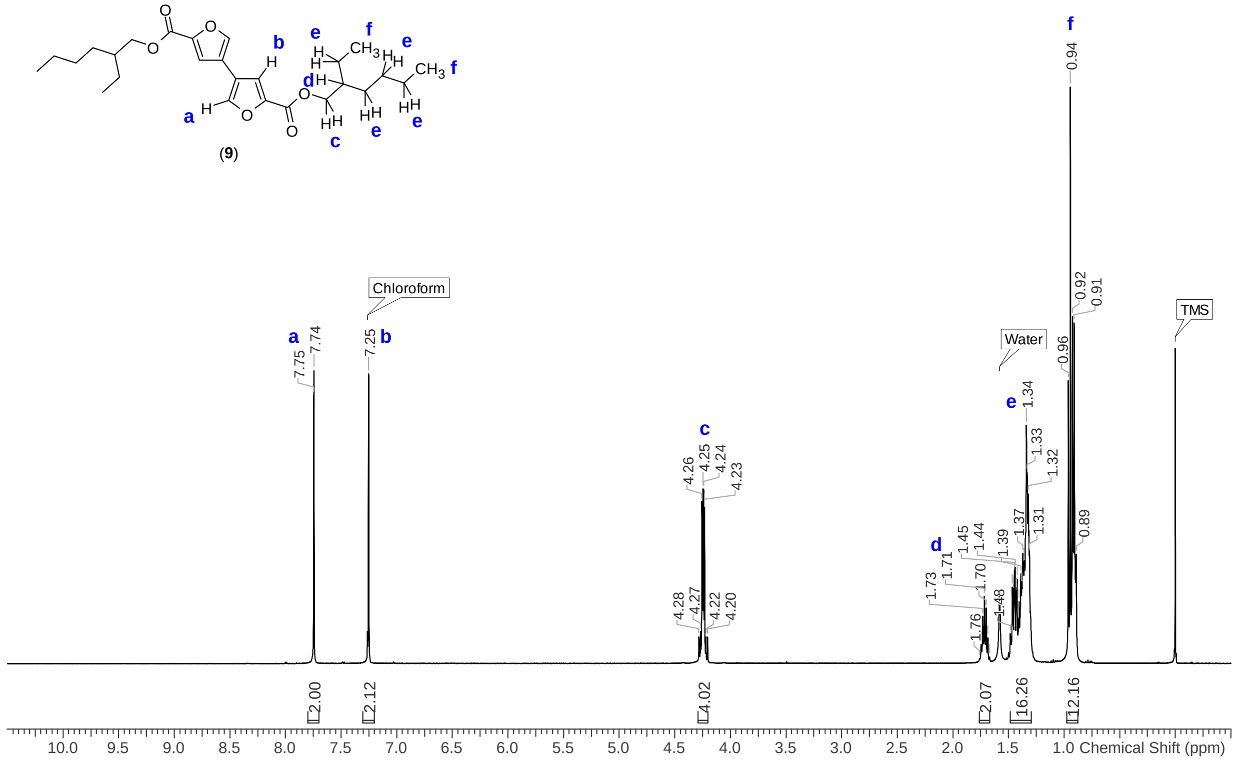

Figure S9. ^{1}H NMR spectrum of **9**.

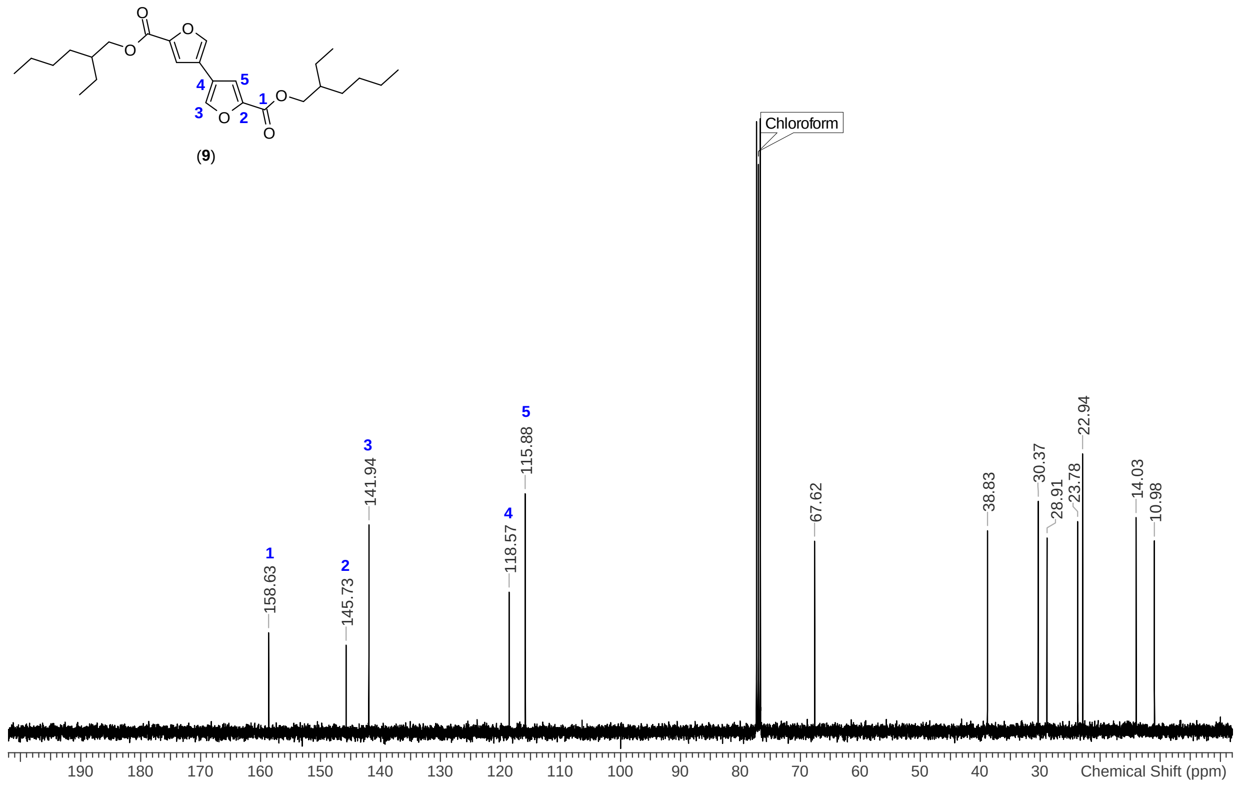

Figure S10. ^{13}C NMR spectrum of **9**.

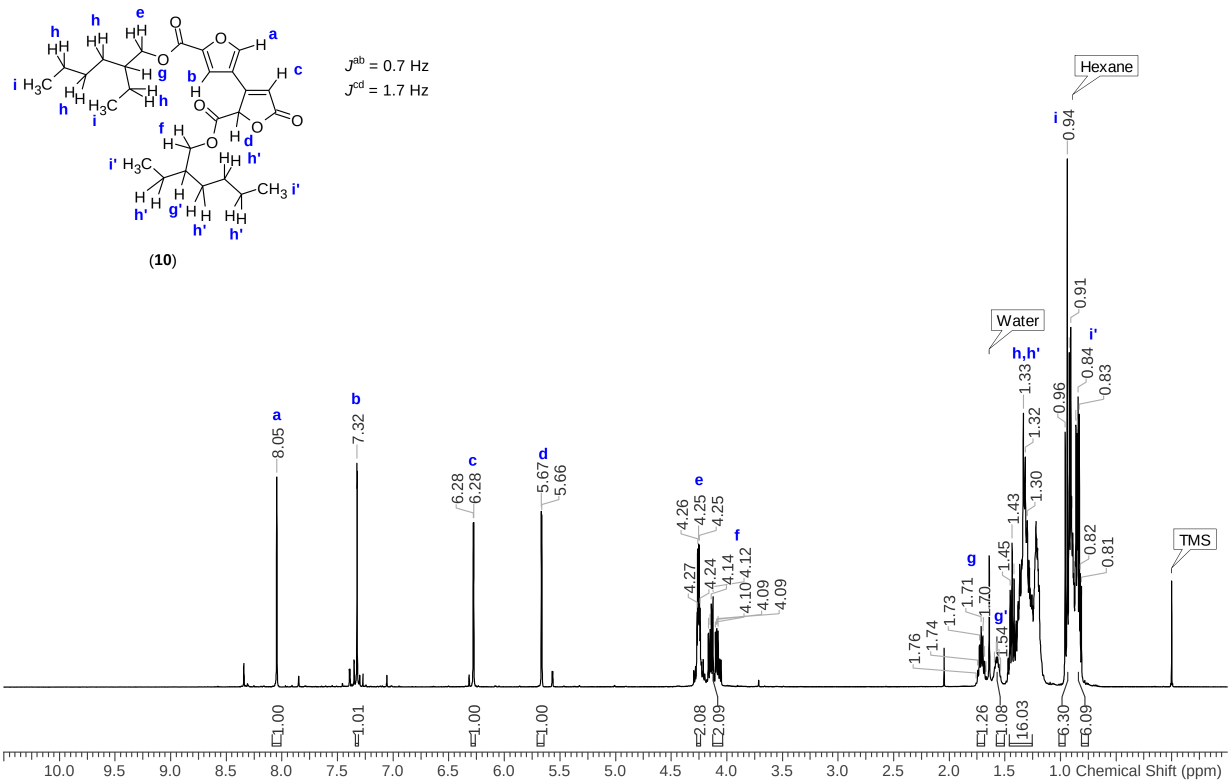

Figure S11. ^{1}H NMR spectrum of **10**.

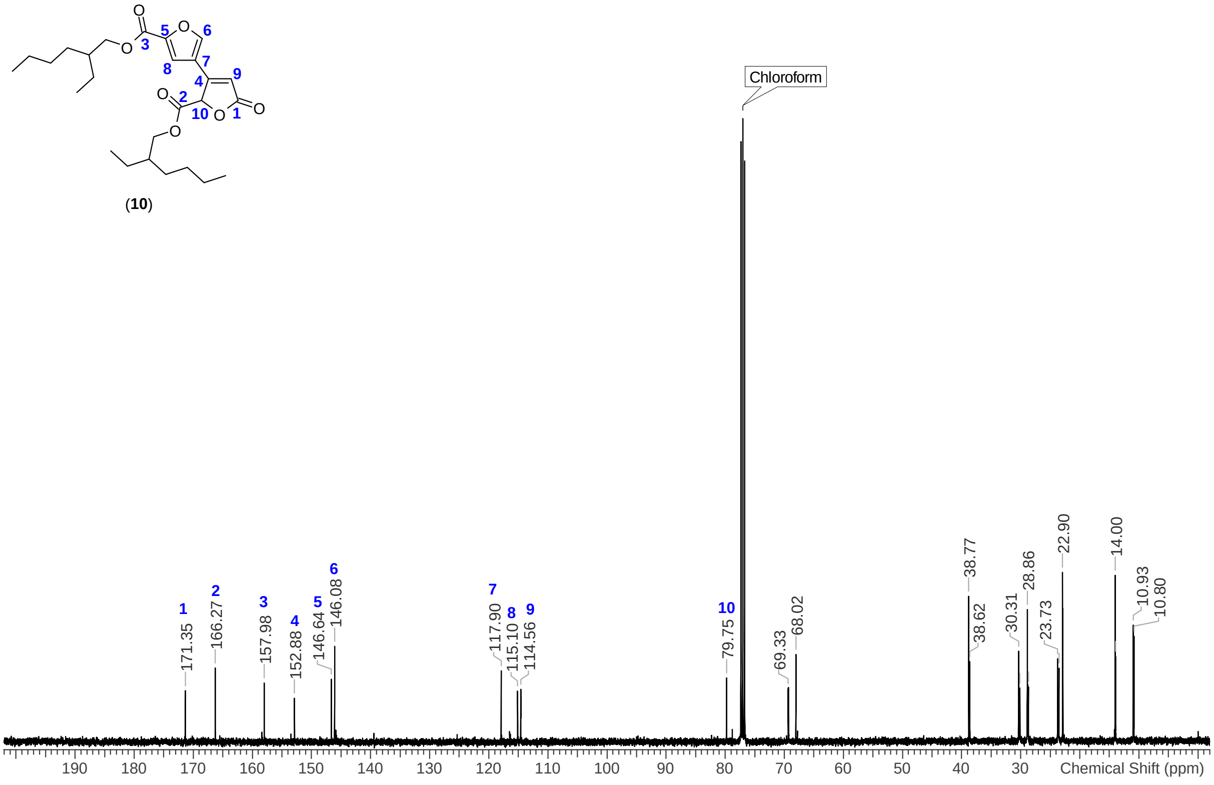

Figure S12. ^{13}C NMR spectrum of **10**.

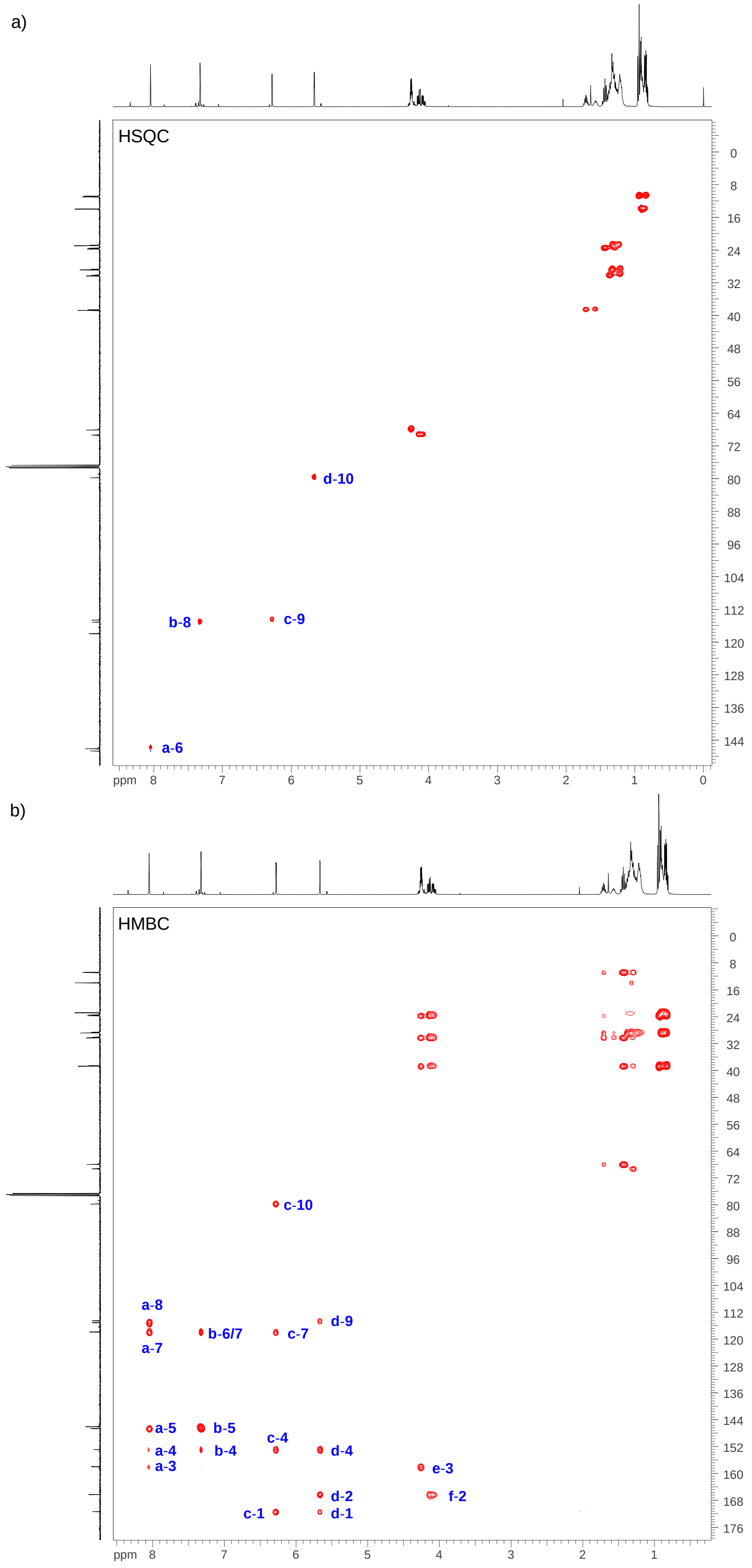

Figure S13. 2D NMR spectra of **10** a) HSQC b) HMBC.

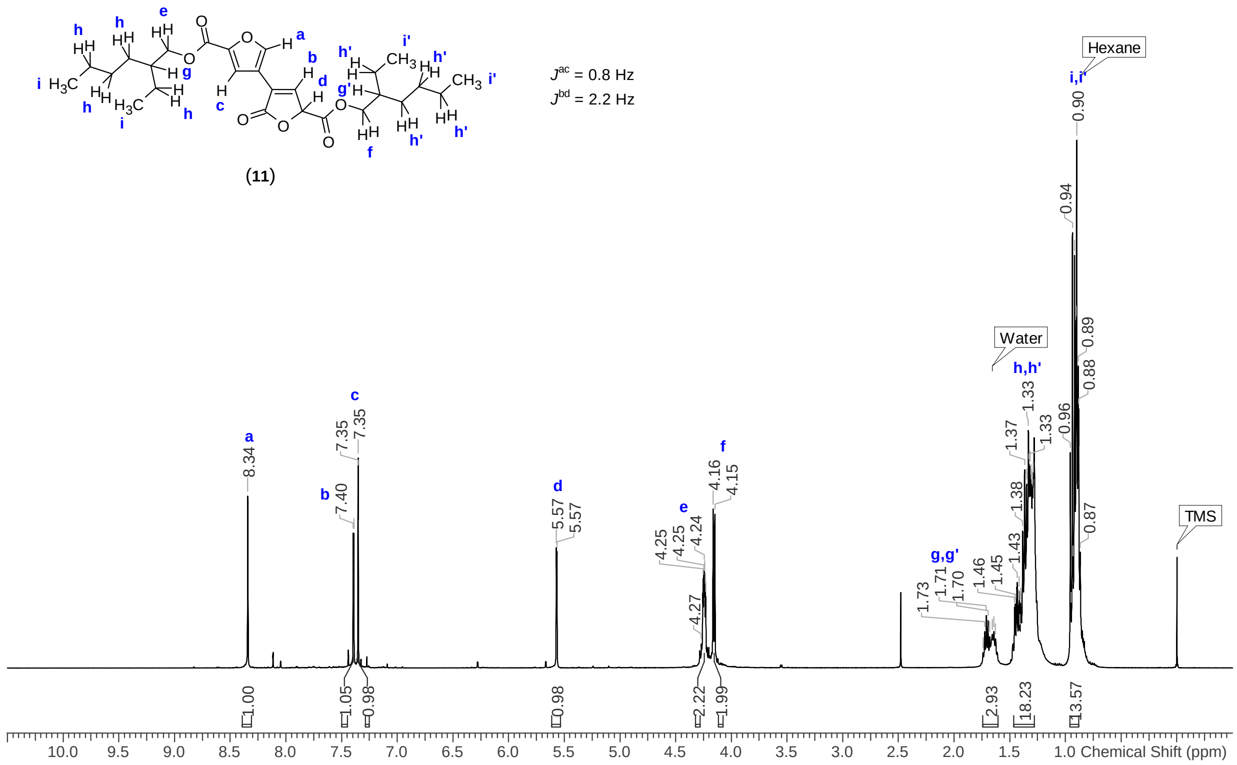

Figure S14. ^{1}H NMR spectrum of **11**.

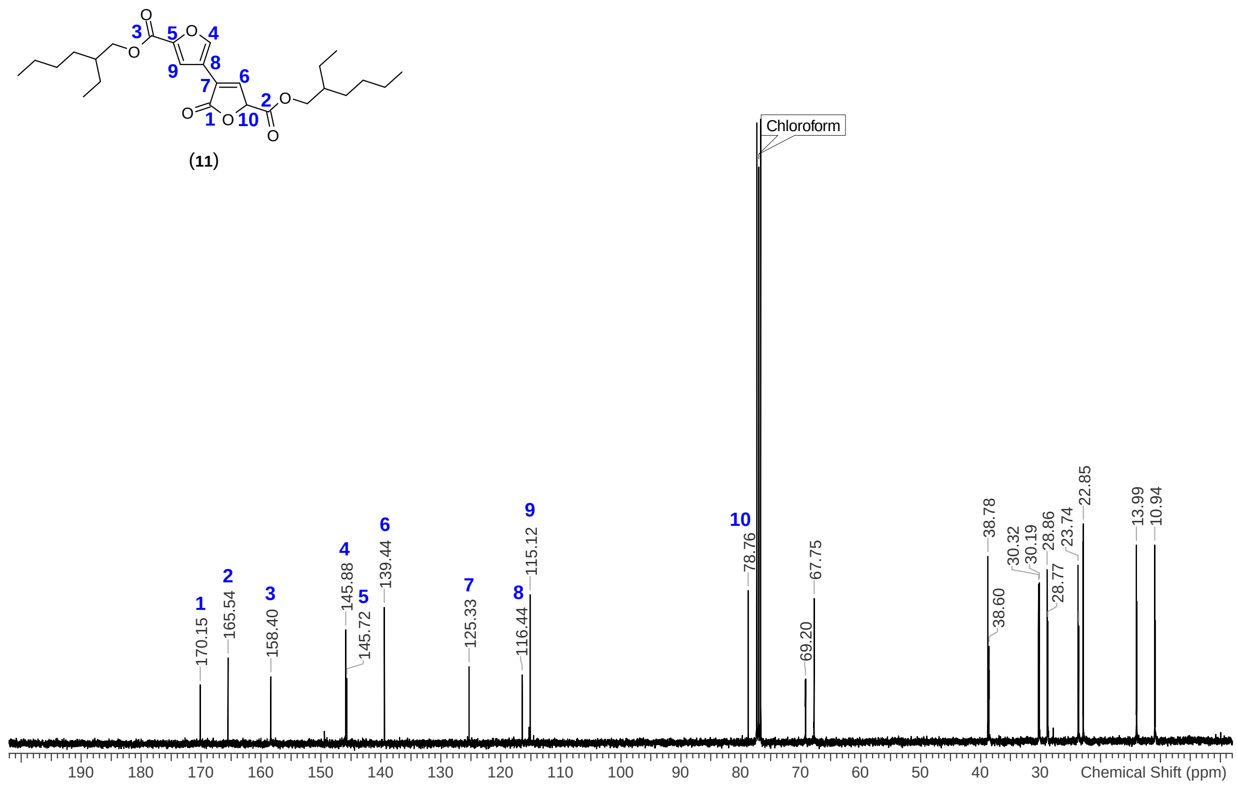

Figure S15. ^{13}C NMR spectrum of **11**.

a)

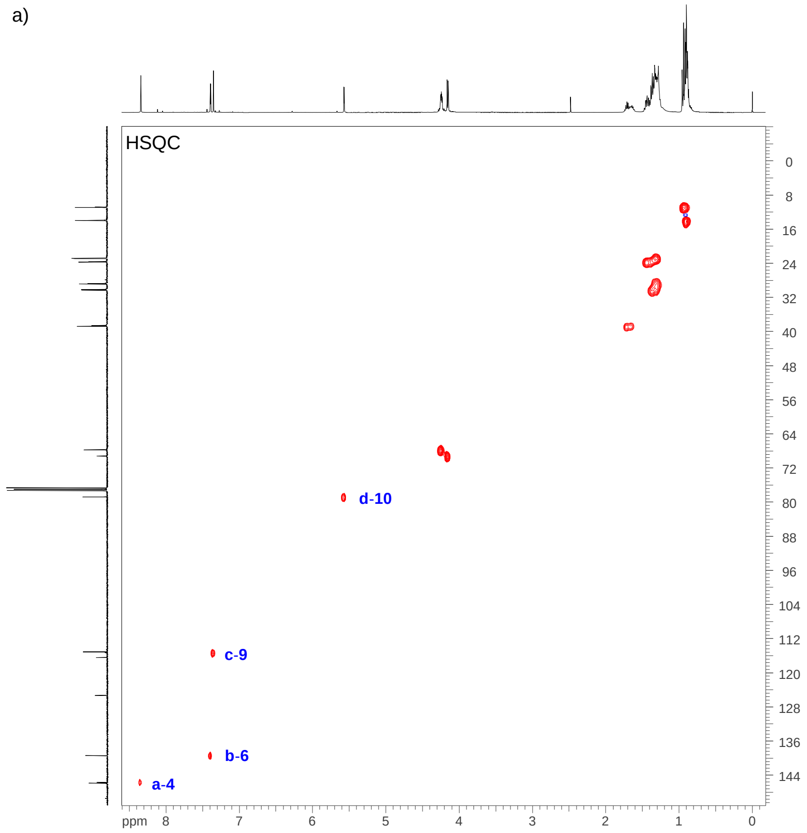

b)

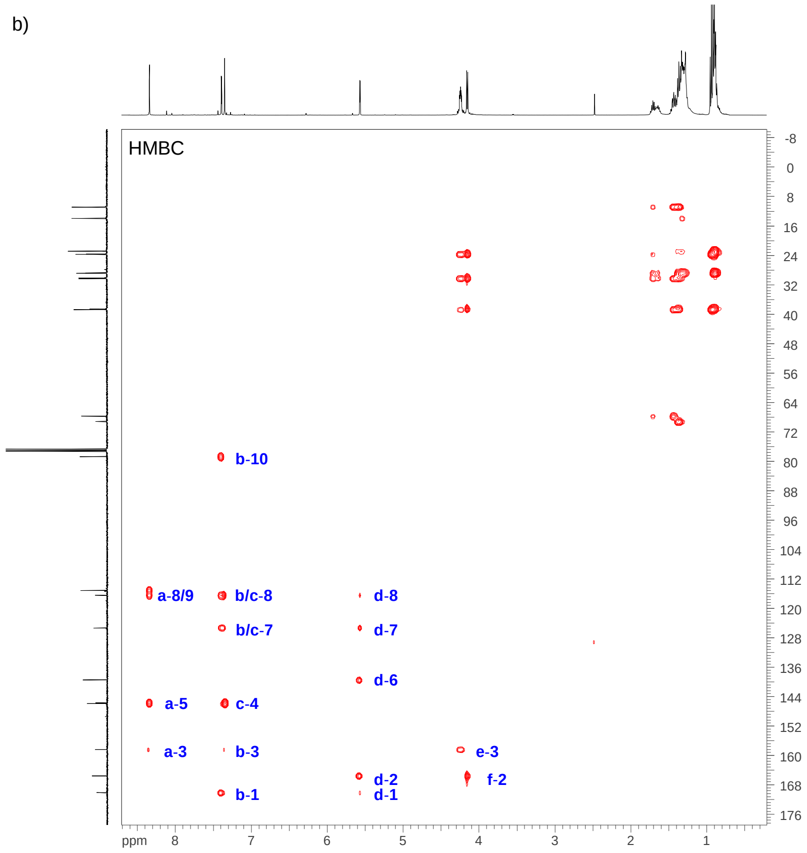

Figure S16. 2D NMR spectra of **11** a) HSQC b) HMBC.

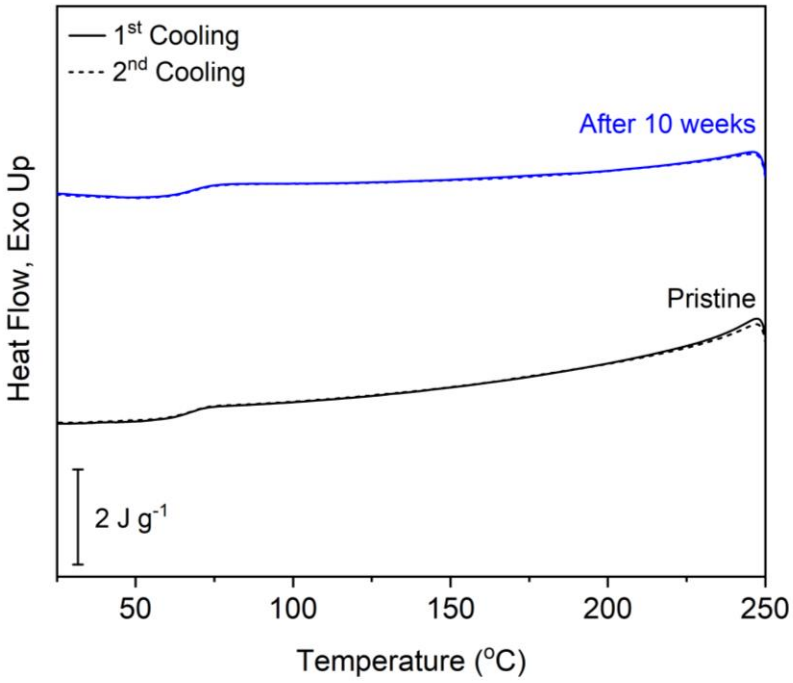

Figure S17. DSC traces from 1st and 2nd cooling of 3,3'-PPeBf.

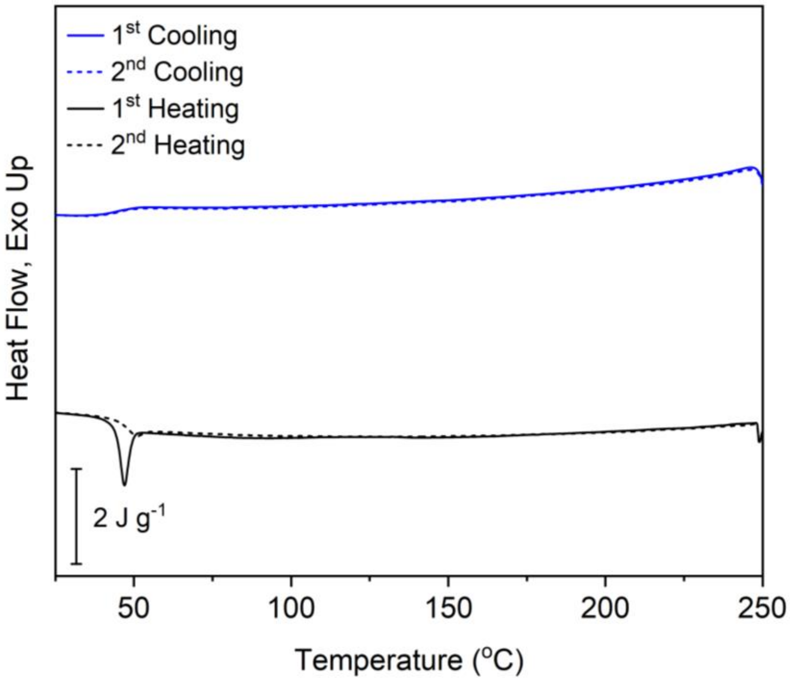

Figure S18. DSC heating and cooling traces for 2,2'-PPeBf.

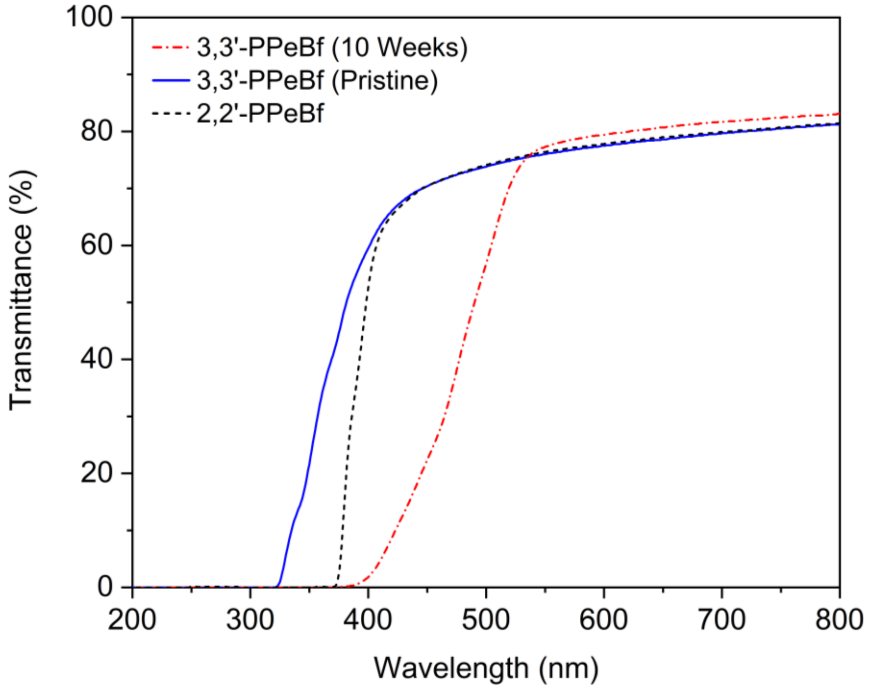

Figure S19. UV-vis transmittance curves of 3,3’-PPeBf and 2,2’-PPeBf films.

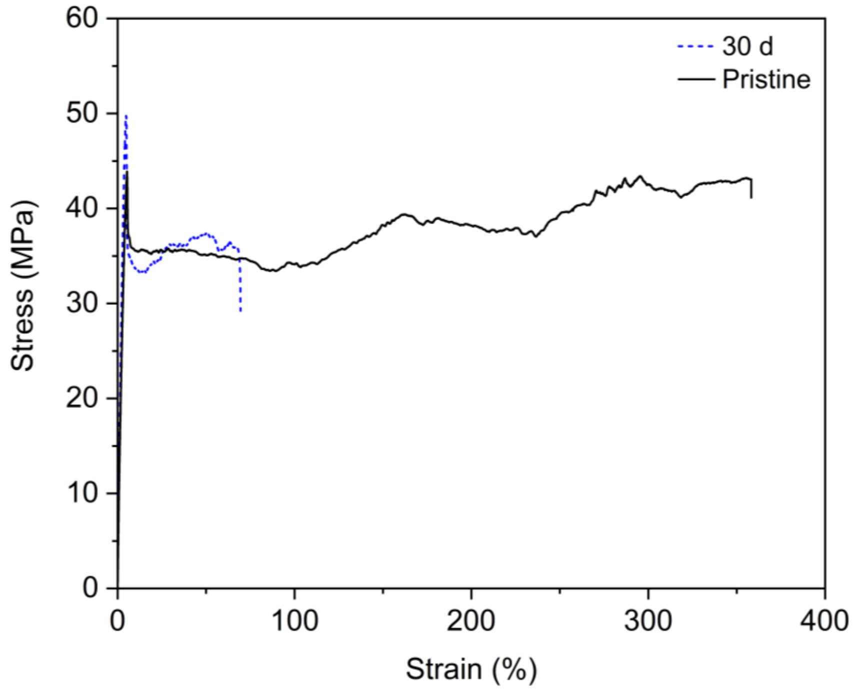

Figure S20. Representative stress-strain curves for pristine and aged 3,3’-PPeBf films.

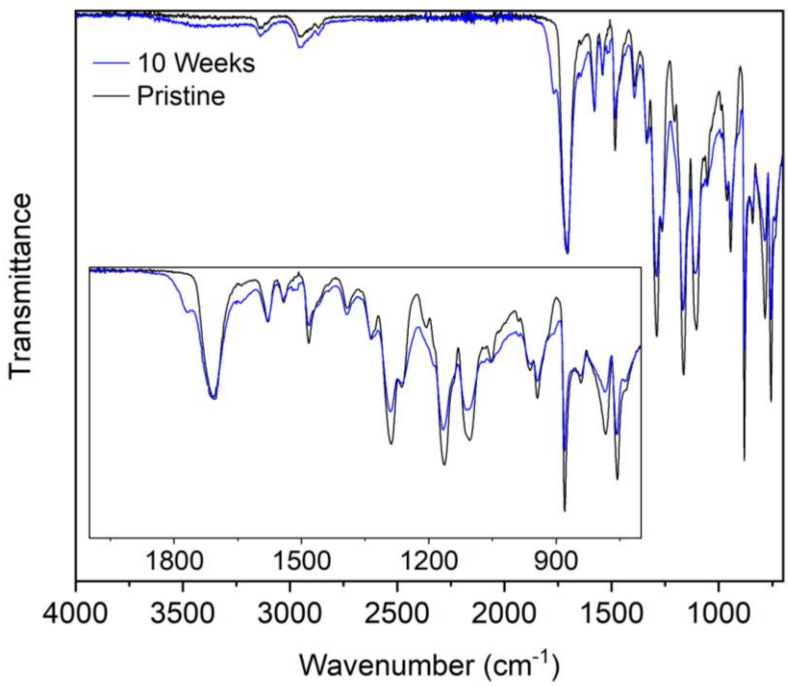

Figure S21. ATR FTIR spectra for 3,3'-PPeBf samples, pristine and air-exposed.

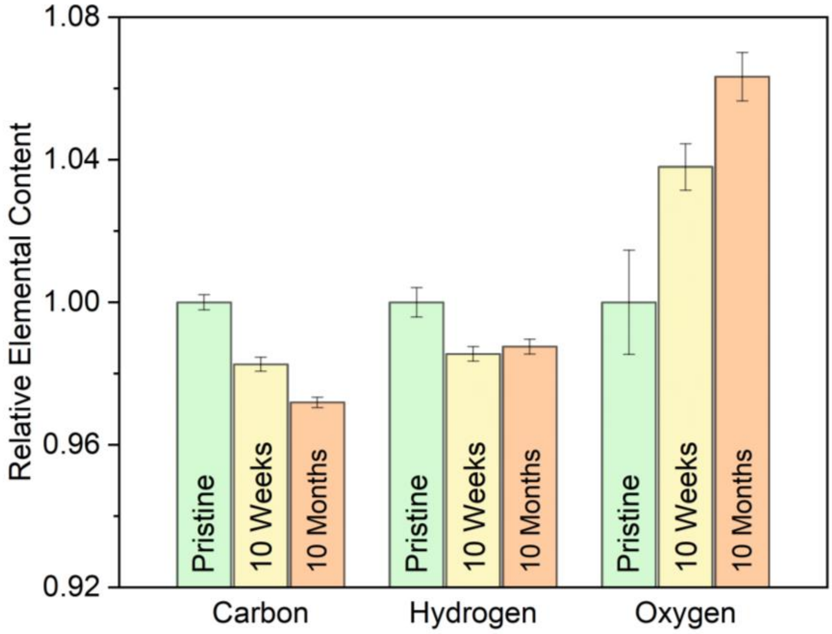

Figure S22. Comparison of elemental analysis results from 3,3'-PPeBf films with different aging times under air.

Table S1. Thermal properties of 3,3'-PPeBf and reported comparable polyesters

Material	T_g (°C) 1st cool	T_g (°C) 2nd heat	T_{cc} (°C) 1st heat	T_{cc} (°C) 2nd heat	T_m (°C) 1st heat	T_m (°C) 2nd heat	ΔH_m (J g^{-1}) 1st heat	ΔH_m (J g^{-1}) 2nd heat	T_c (°C) 1st cool
3,3'-PPeBf[a]	67	67	125	126	182	182	34.7 (31.4)*	32.3 (32.0)**	nd
3,3'-PPeBf[b]	68	69	128	143	178	178	14.4 (14.0)*	8.7 (7.1)**	nd
2,2'-PPeBf	45	46	nd	nd	nd	nd	nd	nd	nd
PPeF[c]		13							
PPeF[d]		24							
PPeT[e]		14		73		132		33.2	62
2,4-PBF[f]		33							

T_g: glass transition temperature. T_{cc}: cold-crystallization temperature. T_m: melting temperature. ΔH_m: melting enthalpy. T_c: crystallization temperature. [a]Pristine, "as-received" film. [b]Film stored for 10 weeks under 50% RH at 23 °C. [c]Ref 2. [d]Ref 3. [e]Ref 4. [f]Ref 5. *Cold-crystallization enthalpy during 1st heat. **Cold-crystallization enthalpy during 2nd heat. nd: not detected.

Table S2. Tensile properties of 3,3'-PPeBf and reported comparable polyesters

Material	E_t (GPa)	σ_y (MPa)	σ_m (MPa)	ε_b (%)	Reference
3,3'-PPeBf[a]	1.38 ± 0.06	43.2 ± 2.0	45.4 ± 3.5	404 ± 104	This work
3,3'-PPeBf[b]	1.42 ± 0.07	47.5 ± 3.6	47.5 ± 3.6	67 ± 19	This work
PPeF	0.009 ± 0.001	-	6 ± 1	1050 ± 200	2
PPeF	0.006 ± 0.001	-	14 ± 2	320 ± 11	3
PPeT[c]	0.65 ± 0.06	22.8 ± 0.7	-	448 ± 31	4
2,4-PBF[d]	0.939 ± 0.072	16 ± 1	-	564 ± 139	5
2,4-PBF[e]	1.330 ± 0.180	35 ± 5	-	208 ± 82	5

E_t: tensile modulus. σ_y: yield stress. σ_m: maximum stress. ε_b: elongation at break. [a]Pristine sample, stored under air at 50% RH and 23 °C for 50 h. [b]Aged sample, stored under air for 30 d at 50% RH and 23 °C. [c]Injection molded specimens. [d]Amorphous samples. [e]Annealed samples.

Table S3. Water contact angles measured in air

Material	WCA (°)
3,3'-PPeBf[a]	93.49 ± 1.44
3,3'-PPeBf[b]	55.69 ± 3.67
2,2'-PPeBf	72.66 ± 2.05
PET	84.73 ± 3.17

WCA: Water contact angle measured in air at 20 °C, mean of left and right contact angles. [a]Pristine, "as-received" film. [b]Film stored under air for at least 10 weeks under 50% RH at 23 °C

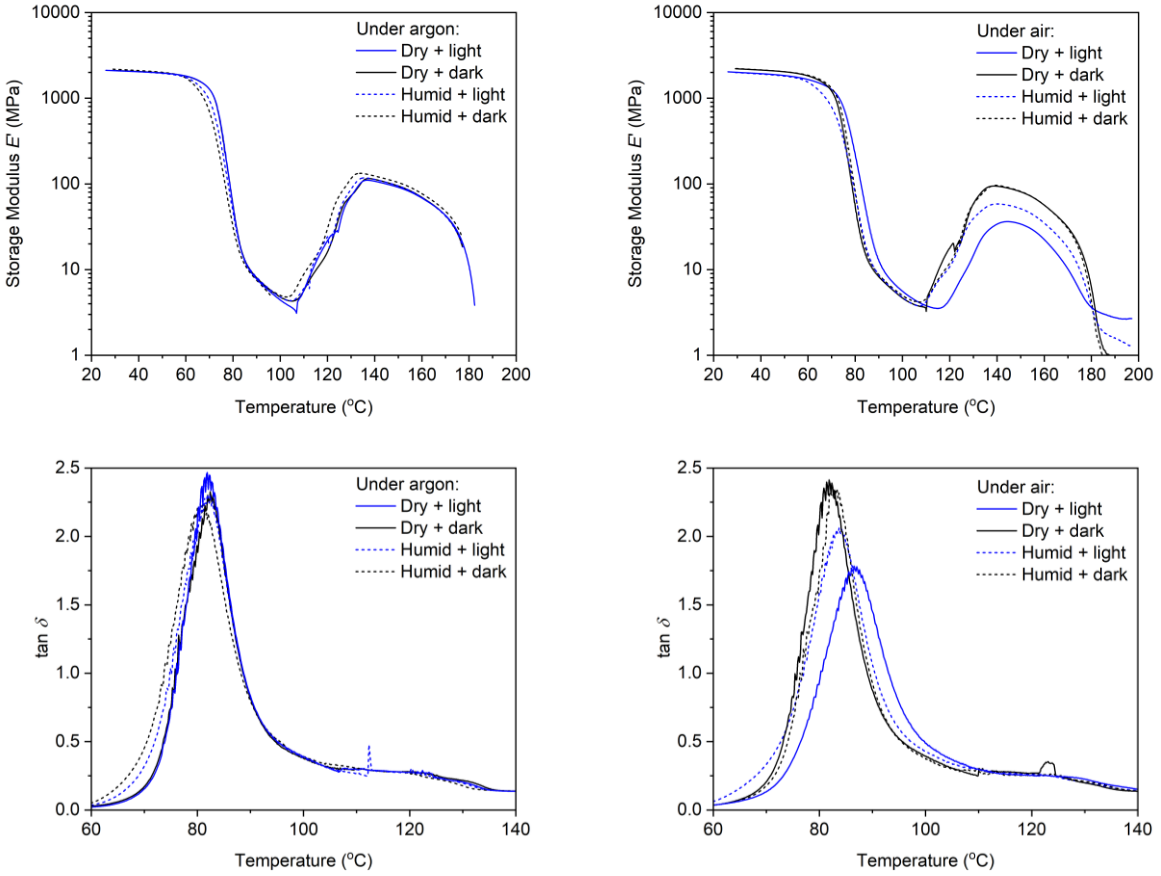

Figure S23. DMA curves from 4-week-old 3,3'-PPeBf film samples after storage at various conditions under air or argon.

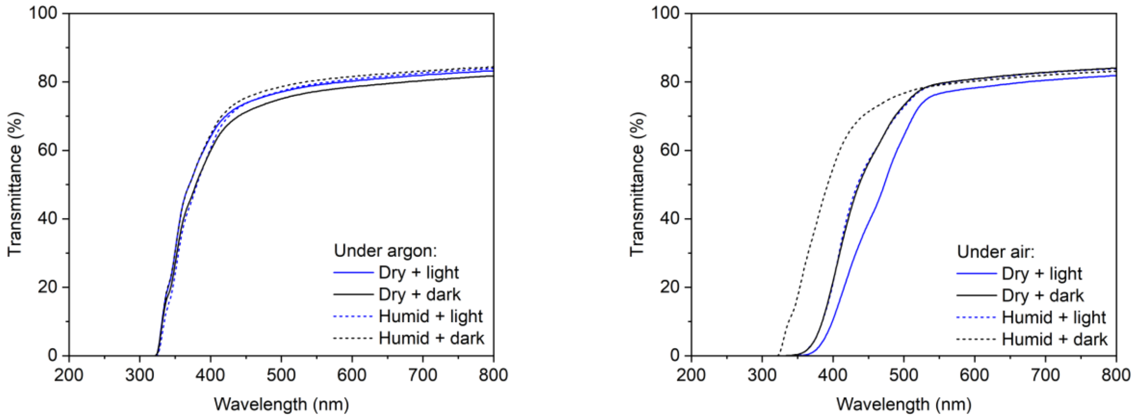

Figure S24. UV-vis curves from 4-week-old 3,3'-PPeBF film samples after storage at various conditions under air or argon.

Table S4. Evaluation of aging factors for 3,3'-PPeBf films via UV-vis and DMA after 4 weeks

Sample storage condition	Cut-off wavelength[a] (nm)	50% transmission cut-off[b] (nm)	Storage modulus peak value[c] (MPa)	T_g from peak of tan δ (°C)
A (dry air, dark)	333	436	94	82
B (humid air, dark)	321	392	96	83
C (dry air, light)	353	472	36	87
D (humid air, light)	339	434	58	84
E (dry argon, dark)	321	379	116	82
F (humid argon, dark)	322	370	133	81
G (dry argon, light)	322	369	111	82
H (humid argon, light)	323	380	118	82

[a]Wavelength below which transmission was <0.1%. [b]Wavelength below which transmission was <50%.
[c]Maximum storage modulus detected after 100 °C during DMA.

Discussion: The storage modulus peak value after the onset cold-crystallization (ca. 105 °C) for 3,3'-PPeBf is affected by cross-linking, which hinders crystallization and results in lower peak value (Fig. 23, Table S4). Simultaneously, the peak of tan δ shifts towards higher temperatures. For samples stored under argon at different conditions, the storage moduli after the onset of cold-crystallization peaked between 111–133 MPa. For samples stored under air, the peaks occurred between 36–96 MPa, with both samples stored under dark (humid or dry) having similar peak values. The sample stored in dry conditions under ambient light had the lowest value at 36 MPa, while the similar sample stored in humid conditions had a higher peak value of 58 MPa. The corresponding trends were observer for the peak temperature of tan δ. In other words, the absence of light slowed down the cross-linking, as did high humidity. Under argon, both incident light and humidity did not appear to have any notable effects, as expected. Cross-linking appeared fastest under dry air and ambient light.

As for the UV-vis measurements (Fig. S24), the sample stored under dry air and ambient light showed the largest change is transmittance, corroborating the results of DMA (Table S4). In contrast, the sample stored under humid air in the dark showed the least yellowing, revealing the accelerating and retarding effects of ambient light and humidity, respectively. Samples stored under argon did not show change in transmittance over the same time period. From these tests, the following conclusions are drawn: 1) O_2 is required for the observed process 2) humidity can slow the process 3) ambient light can accelerate the process.

The model compound aging experiment supported the notion that lactones can be formed when 3,3'-BFDCA derivatives are in contact with air. Isolated compounds **10** and **11** appeared to be isomers, and accurate mass measurements yielded the same molecular weight for both: The mass had increased by the mass of a single oxygen atom. Two structurally related compounds with NMR data are presented in Scheme S1 for comparison (data from references[6,7]; assignment presented is our own) with the above NMR spectra.

Scheme S1. Chemical shifts for the lactone ring in 3-phenylfuran-2(5*H*)-one and 3-phenylfuran-2(5*H*)-one

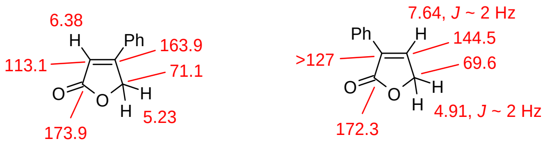

4-Phenylfuran-2(5*H*)-one[6] 3-Phenylfuran-2(5*H*)-one[7]

Good matches for the substituent pattern were not available in the literature (and therefore exact chemical shift matches), but the relative differences between the isomers are consistent when compared with those of **10** and **11**. The mechanisms by which the products form are still unclear, but the mechanism could involve the initial formation of a 2-hydroxyfuran intermediate via oxidation (Scheme S2).

Scheme S2. Possible pathways to unsaturated lactone products from 3,3'-bifurans

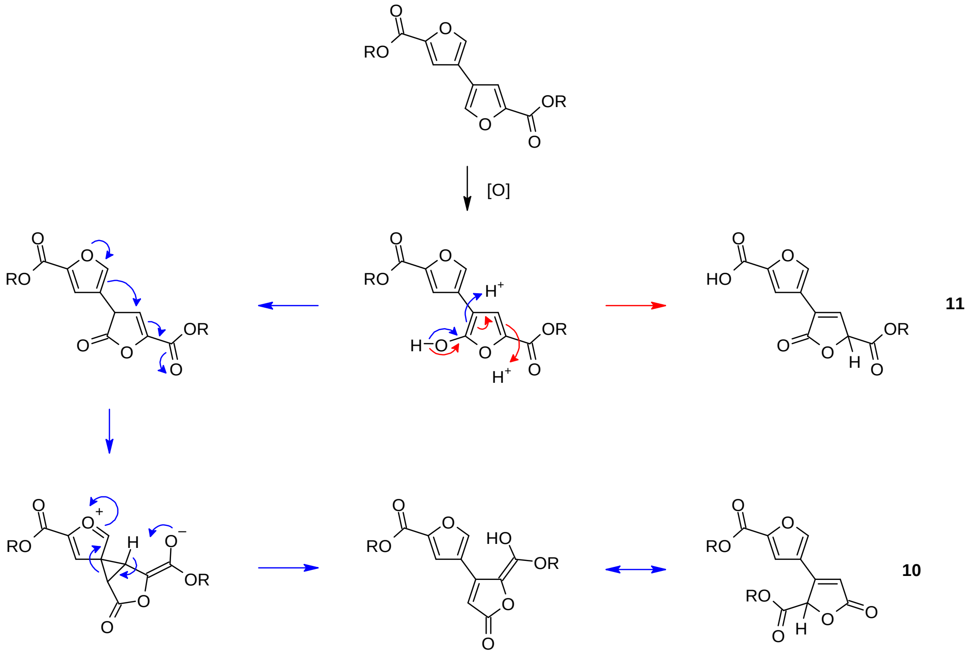

11

10

The initial oxidized intermediate may form due to hydrogen atom abstraction by O_2, possibly involving participation from another bifuran that accepts the second oxygen atom. An alternative explanation might be an initial reaction with singlet oxygen, which is known to easily react with furans of various kinds and provide lactones as products. However, singlet oxygen is normally generated using separate photosensitizers and high-energy light sources (i.e., UV light), both of which were absent here. The tentative structure of **10**, where the furan ring substituent appears to be located on a different position of the lactone ring, is also curious. Since heat was applied to prevent **9** from crystallizing, the room-temperature degradation of the polyester may follow different routes with different end products.

Cross-linked-like structures could not be isolated after the model compound experiment, but it would appear feasible for the cross-linking to occur due to these types of reactive lactone intermediates. They should result in the build-up of both new carbonyls and new conjugated moieties, increasing the polarity and yellowness of 3,3'-PPeBf. Hydrolysis experiments also revealed a link between the polyester and the model compounds:

When 3,3-PPeBf (solvent precipitated) with extensive air-exposure (6 months) was hydrolyzed under reflux with excess aqueous NaOH, acidification allowed the separation of pure 3,3'-BFDCA in reasonable yield (ca. 60%) via filtration. In addition to 3,3'-BFDCA, the acidic filtrate contained a complex mix of products that was extracted into ethyl acetate and analyzed using ^{1}H NMR (Fig. S25). It is notable that the spectrum measured from this mixture contains key similarities with the hydrolysate mixture obtained from **11** under similar conditions. This reinforces the notion that the model compound experiments and air-aging of the polyester share reaction pathways.

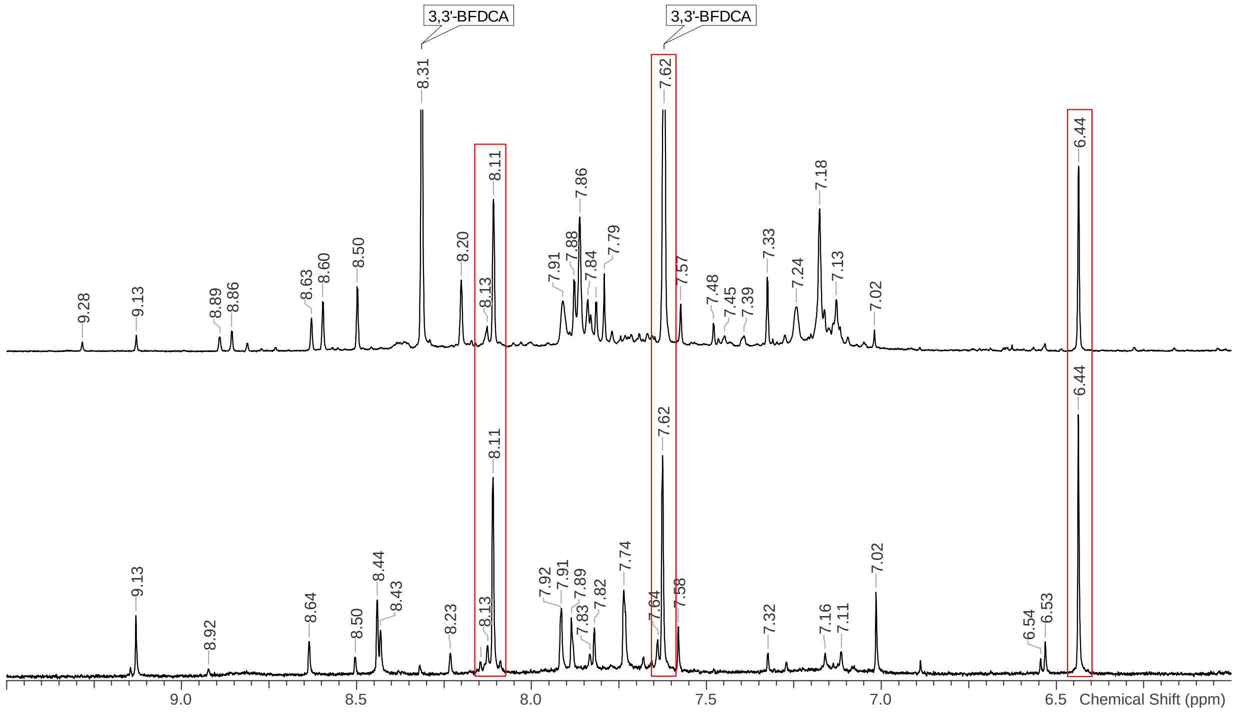

Figure S25. ^{1}H NMR spectra (6–9.5 ppm) measured in $(CD_3)_2SO$ from the hydrolysate of **11** (bottom) and the hydrolysate side-fraction of air-aged 3,3'-PPeBf (top).

References

1. Billmeyer, F. Methods for estimating intrinsic viscosity. *J. Polym. Sci.* **1949**, *4*, 83–86.

2. Guidotti, G.; Soccio, M.; García-Gutiérrez, M. C.; Ezquerra, T.; Siracusa, V.; Gutiérrez-Fernández, E.; Munari, A.; Lotti, N. Fully Biobased Superpolymers of 2,5-Furandicarboxylic Acid with Different Functional Properties: From Rigid to Flexible, High Performant Packaging Materials. *ACS Sustainable Chem. Eng.* **2020**, *8*, 9558–9568.

3. Xie, H.; Wu, L.; Li, B.-G.; Dubois, P. Modification of Poly(ethylene 2,5-furandicarboxylate) with Biobased 1,5-Pentanediol: Significantly Toughened Copolyesters Retaining High Tensile Strength and O_2 Barrier Property. *Biomacromolecules* **2019**, *20*, 353–364.

4. Lu, J.; Zhou, L.; Xie, H.; Wu, L.; Li, B.-G. Biobased flexible aromatic polyester poly(1,5-pentylene terephthalate) (PPeT): Revisiting melt crystallization behaviors and thermo-mechanical properties. *Eur. Polym. J.* **2019**, *110*, 168–175.

5. Bianchi, E.; Soccio, M.; Siracusa, V.; Gazzano, M.; Thiyagarajan, S.; Lotti, N. Poly(butylene 2,4-furanoate), an Added Member to the Class of Smart Furan-Based Polyesters for Sustainable Packaging: Structural Isomerism as a Key to Tune the Final Properties. *ACS Sustainable Chem. Eng.* **2021**, *9*, 11937–11949.

6. Yu, S., Hong, C., Liu, Z., Zhang, Y. Cobalt-Catalyzed Vinylic C–H Addition to Formaldehyde: Synthesis of Butenolides from Acrylic Acids and HCHO. *Org. Lett.* **2021**, *23*, 8359–8364.

7. Gronnier, C., Kramer, S., Odabachian, Y., Gagosz, F. Cu(I)-Catalyzed Oxidative Cyclization of Alkynyl Oxiranes and Oxetanes. *J. Am. Chem. Soc.* **2012**, *134*, 828–83.
